# DNA methylation signatures of adolescent victimization: analysis of a longitudinal monozygotic twin sample

**DOI:** 10.1080/15592294.2020.1853317

**Published:** 2020-12-29

**Authors:** Radhika Kandaswamy, Eilis Hannon, Louise Arseneault, Georgina Mansell, Karen Sugden, Benjamin Williams, Joe Burrage, James R Staley, Ehsan Pishva, Aisha Dahir, Susanna Roberts, Andrea Danese, Jonathan Mill, Helen L Fisher, Chloe C. Y. Wong

**Affiliations:** a King’s College London, Social, Genetic & Developmental Psychiatry Centre, Institute of Psychiatry, Psychology & Neuroscience, London, UK; b University of Exeter Medical School, University of Exeter, Exeter, UK; c ESRC Centre for Society & Mental Health, King’s College London, London, UK; d Department of Psychology and Neuroscience, Duke University, Durham, NC, USA; e MRC Integrative Epidemiology Unit, Bristol Medical School, University of Bristol, Bristol, UK; f King’s College London, Department of Psychology, Institute of Psychiatry, Psychology & Neuroscience, London UK; g King’s College London, Department of Child & Adolescent Psychiatry, Institute of Psychiatry, Psychology & Neuroscience, London UK; h National & Specialist CAMHS Clinic for Trauma, Anxiety and Depression, South London & Maudsley NHS Foundation Trust, London, UK

**Keywords:** Adolescence, adversity, DNA methylation, epigenetics, longitudinal, twins, victimization

## Abstract

Accumulating evidence suggests that individuals exposed to victimization at key developmental stages may have different epigenetic fingerprints compared to those exposed to no/minimal stressful events, however results are inconclusive. This study aimed to strengthen causal inference regarding the impact of adolescent victimization on the epigenome by controlling for genetic variation, age, gender, and shared environmental exposures. We conducted longitudinal epigenome-wide association analyses (EWAS) on DNA methylation (DNAm) profiles of 118 monozygotic (MZ) twin pairs from the Environmental Risk study with and without severe adolescent victimization generated using buccal DNA collected at ages 5, 10 and 18, and the Illumina EPIC array. Additionally, we performed cross-sectional EWAS on age-18 blood and buccal DNA from the same individuals to elucidate tissue-specific signatures of severe adolescent victimization. Our analyses identified 20 suggestive differentially methylated positions (DMPs) (*P* < 5e-05), with altered DNAm trajectories between ages 10–18 associated with severe adolescent victimization (*∆Beta range *= −5.5%−5.3%). Age-18 cross-sectional analyses revealed 72 blood (*∆Beta range *= −2.2%−3.4%) and 42 buccal (*∆Beta range *= −3.6%−4.6%) suggestive severe adolescent victimization-associated DMPs, with some evidence of convergent signals between these two tissue types. Downstream regional analysis identified significant differentially methylated regions (DMRs) in *LGR6* and *ANK3* (Šidák *P = 5*e-09 and 4.07e-06), and one upstream of *CCL27* (Šidák *P = *2.80e-06) in age-18 blood and buccal EWAS, respectively. Our study represents the first longitudinal MZ twin analysis of DNAm and severe adolescent victimization, providing initial evidence for altered DNA methylomic signatures in individuals exposed to adolescent victimization.

## Introduction

Exposure to stress during childhood and adolescence is detrimental to adult health and findings from a number of studies have linked early-life stress with a range of psychiatric and physical disorders that persist into adulthood [1–3]. A recent retrospective survey conducted by the World Health Organization reported that nearly 40% of adults experienced some form of severe stress during childhood and/or adolescence [4]. However, there is evidence of high inter- and intra-individual variability and adaptability in the stress response system resulting from a complex interaction between multiple genes and the social environment [5,6]. Although there is accumulating evidence suggesting that exposure to early-life stress, including victimization, leads to adverse outcomes in later life [7,8], the potential mechanisms underlying the ‘biological embedding’ of these psychosocial experiences are less well understood. One possibility is that environmental conditions could affect or interact with genes through epigenetic mechanisms, including DNA methylation (DNAm), which may mediate long-term effects on health. DNA methylation can alter the way in which genes are expressed without inducing changes in the actual sequence of the genes, thereby, having functional consequences [9].

Epigenetic processes are dynamic and can fluctuate across the lifespan in response to genetic and environmental influences, especially during key developmental periods such as early childhood and adolescence [10]. However, some epigenetic patterns especially those required for cell-lineage classification may be retained as a form of epigenetic memory [11]. There has been a steady rise in studies linking victimization exposure (such as physical and sexual abuse, neglect, and bullying by peers) to changes in DNAm [12–15] but definitive evidence is lacking due to diverse study populations including different ages and ethnic backgrounds, inconsistent methodology and non-overlapping results. Many of the studies conducted to date adopted a cross-sectional, case-control design which do not account for changes in DNA methylation over time, nor the underlying genetic differences, known to affect liability to stress reactivity and its interaction with victimization exposure as well as epigenetic processes [14,16,17]. Some of these findings might also be confounded by potential recall and recruitment bias, e.g. the use of adult retrospective reports of stress or trauma, unusual clinical groups such as suicide victims or institutionalized children, or relatively small sample sizes [18–22]. Moreover, childhood victimization has been the focus of the majority of these studies with very few investigating the association between victimization in adolescence, a potentially key sensitive window for long-term physiological and behavior changes [23,24] in which victimization exposures peak [25] and altered epigenetic profiles have been reported [14,26,27]. Adolescence is also an important developmental phase to focus upon because the majority of individuals who experience severe mental health problems develop them during this period [28] and many of these have been associated with exposure to victimization in adolescence [29–31].

To this end, we performed a genetically-informed epigenome-wide association study (EWAS) to explore the impact of severe victimization during adolescence on the epigenome by combining the monozygotic (MZ) twin design with a longitudinal approach [32,33]. Also, to isolate epigenetic patterns associated with adolescent victimization we purposely selected twins where neither twin had been exposed to severe victimization during childhood (that is not exposed to severe physical, sexual or emotional abuse, bullying by peers, neglect, or domestic violence by age 12 years). In our main analyses, we conducted a longitudinal EWAS using methylation data from buccal DNA collected from the same individual before and after severe adolescent victimization exposure and compared these profiles to those of the unexposed twins. This enabled us to explore the longitudinal epigenetic trajectories associated with severe adolescent victimization and minimized the potentially confounding effects of genetic variation, age, sex, and shared environmental exposures that are common limitations of previous epigenetic studies. We also performed parallel EWAS in the blood and buccal samples obtained from the same individuals at age 18 and explored the potential tissue-specific epigenetic signatures associated with severe adolescent victimization. Finally, taking advantage of the discordant MZ twin design, we conducted an exploratory analysis of within-twin-pair methylation changes where one twin in each pair had been exposed to severe adolescent victimization, while the other had not, to more stringently control for unmeasured shared environmental and genetic factors.

## Materials and methods

### Study cohort

Participants were members of the Environmental Risk (E-Risk) Longitudinal Twin Study described in detail previously [34] and in the Supplementary Methods. Briefly, the E-Risk study tracks the development of a 1994–1995 birth cohort of 2,232 British children. The study sample was constructed in 1999 and 2000, when 1,116 families (93% of those eligible) with same-sex 5-year-old twins participated in home-visit assessments. This sample comprised 56% monozygotic (MZ) and 44% dizygotic (DZ) twin pairs, and sex was evenly distributed within zygosity (49% male). Home visits were conducted when participants were aged 5, 7, 10, 12, and 18 years with 93% retention. There were no differences between those who did and did not take part at age 18 in terms of socioeconomic status (SES) assessed when the cohort was initially defined (χ^2^ = 0.86, *p* = 0.65), age-5 IQ scores (*t* = 0.98, *p* = 0.33), or age-5 internalizing or externalizing behavior problems (*t* = 0.40, *p* = 0.69 and *t* = 0.41, *p* = 0.68, respectively). The Joint South London and Maudsley and the Institute of Psychiatry Research Ethics Committee approved each phase of the study. Parents gave informed consent, and participants gave assent at ages 5–12 and informed consent at age 18.

### Victimization exposure

Childhood and adolescent victimization experiences in this cohort have been described previously [35,36] and are summarized briefly here. Full details are provided in the Supplementary Methods.

### Childhood victimization (0-12 years)

Exposure to childhood victimization since birth was assessed repeatedly when the children were 5, 7, 10, and 12 years old, including exposure to violence between the mother and her partner, frequent bullying by peers, physical maltreatment by an adult, sexual abuse, emotional abuse and neglect, and physical neglect. Each exposure across childhood was coded on a 3-point scale (0 = no exposure, 1 = probable/less severe exposure, 2 = definite/severe exposure). All our study participants were selected for having no severe victimization exposure by age 12.

### Adolescent victimization (12-18 years)

At age 18, each twin was interviewed separately about exposure to a range of victimization experiences between ages 12 and 18 using the Juvenile Victimization Questionnaire (JVQ) [37,38] adapted as a clinical interview [36]. Seven forms of victimization were assessed: maltreatment, neglect, sexual victimization, family violence, peer/sibling victimization, cyber-victimization, and crime victimization. Each of these was rated as 0 (no exposure), 1 (some exposure), or 2 (severe exposure) by trained raters based on the descriptions of the experiences provided by participants and using the coding system of the Childhood Experience of Care and Abuse interview manual [39]. Only those with a score of 2 for at least one type of victimization were considered to have been exposed to severe adolescent victimization.

Three groups of MZ twin pairs were selected for the current epigenetic study: *a)* Group 1: discordant MZ twin pairs where only one twin in the pair had reported severe adolescent victimization (N = 62), *b)* Group 2: concordant unexposed MZ twin pairs where both twins had reported no severe adolescent victimization (N = 28), and *c)* Group 3: concordant exposed MZ twin pairs where both twins had reported severe adolescent victimization (N = 28) (Supplementary Table 1).

### Genome-wide DNA methylation analysis

Buccal samples were collected from participants at ages 5, 10 and 18 and whole blood was collected at age 18. Genomic DNA was extracted using standard protocols [40–42]. 500ng of buccal and blood DNA was treated with sodium bisulphite using the EZ96 DNA Methylation kit (Zymo Research, Irvine, California) following the manufacturer’s standard protocol. Repeated samples from twins and their co-twin, including blood and buccal DNA, were processed on the same 96-well plate, and twin pairs belonging to different categories were randomized to minimize potential batch effects. DNAm was assessed using the Illumina Infinium HumanMethylationEPIC BeadChip kit (Illumina, Inc., San Diego, California) and quantified on an Illumina HiScan System (Illumina, Inc.). The level of methylation is expressed as a ‘beta’ value (β-value), ranging from 0 (no cytosine methylation) to 1 (complete cytosine methylation).

All data pre-processing and downstream statistical analyses were performed using R version 3.4.3 [43]. Data quality control (QC) of the methylation profiles is detailed in Supplementary Methods. After stringent QC, the final dataset comprised 736/944 (80%) samples (see details in Supplementary Table 1) and 695,834 probes for downstream statistical analyses. Cell-type composition was estimated using the Houseman algorithm [44] in the blood samples and EPiDISH package [45] in the buccal samples to adjust for the potential differential cellular heterogeneity. Age-18 smoking pack-year data were used as covariates in all relevant analyses. QQplots and regional Manhattan plots were generated using the R packages *qqman* [46] and *ggplot2*. The dataset is accessible from the Gene Expression Omnibus database (accession number: GSE154566).

### Statistical analyses

In this study, we investigated the possible associations between severe adolescent victimization exposure and differential DNA methylation using two statistical models, an *unpaired* main analysis where all individuals (groups 1, 2 and 3) were treated as singletons to maximize power while adjusting for their relatedness structure in the dataset, and a *paired* secondary analysis where we studied the twin intra-pair differences (see Supplementary Figure 1).

## Main analyses

### Longitudinal EWAS using buccal DNA

Methylation β-values from all three time-points (ages 5, 10 and 18) from all the MZ twins in each of the groups (groups 1, 2 and 3, N = 501) (Supplementary Table 1, Supplementary Figure 1a) were treated as singletons whilst adjusting for their relatedness structure in the dataset and modelled over time using linear regression with clustered robust standard errors to account for the non-independence of twin observations [47]. The model was fitted individually for each CpG, with severe adolescent victimization as the exposure of interest and DNAm as the outcome with age, gender, cell-type proportions and smoking status (smoking pack-years at age 18) as covariates. An interaction term for age and severe adolescent victimization was included to dissect the specific effect of exposure on methylation change during childhood (5–10 years) or adolescence (10–18 years).

A simplified version of the model formula is:

DNA methylation ~ Victimization exposure + sex + age + smoking status + cell types + victimization exposure * age, cluster = FamilyID

We used an EPIC array experiment-wide significance threshold of 9e-08 [48] and a suggestive significance *P*-value threshold of *P* < 5e-05 to identify DMPs associated with severe adolescent victimization (i.e. between ages 10 and 18) in this unpaired analysis.

### Cross-sectional EWAS using age-18 blood and buccal DNA

We also performed parallel EWASs to identify potential severe adolescent victimization-associated DNAm variation in age-18 blood and buccal samples using all twins in groups 1, 2 and 3 treated as singletons (Supplementary Figure 1a) whilst adjusting for their relatedness structure in the dataset using linear regression with clustered robust standard errors [47]. Both the models included gender, cell-type proportions and smoking pack years as covariates. The formula is as described here:

#### DNA methylation ~ Victimization exposure + sex + smoking status + cell types, cluster = FamilyID

Similar to the longitudinal analysis, a suggestive *P*-value threshold of *P* < 5e–05 and an EPIC array significance threshold of *P* < 9e–08 were used to identify potential DMPs associated with severe adolescent victimization. We also performed additional exploratory analyses to check for the robustness of the age-18 blood EPIC array data using matched 450 K array data (see Supplementary Methods).

### Exploratory analyses

We capitalized on the availability of twin level data within our study to perform an exploratory paired analysis to identify longitudinal methylation change as a result of severe adolescent victimization by including EWAS data from only complete discordant MZ twin pairs at age 10 and 18 (i.e. group 1, n = 24) (Supplementary Figure 1b). The major advantage of this is that it allows us to fully control for genetic and unmeasured shared environmental influences. Briefly, intra-individual changes in buccal DNAm from ages 10 to 18 were calculated (longitudinal ∆β) and the difference in the longitudinal ∆β between the exposed twin and their unexposed co-twin was examined using a paired *t*-test. We used the established ranked magnitude-significance method [49,50] for the identification of differentially methylated probes. In brief, CpGs were ranked separately using paired *t*-test *P*-value (significance) and the magnitude of the difference in DNAm change (absolute ∆β) and a final ranked list was determined by adding the two ranks. For the cross-sectional analyses, paired *t*-tests were performed separately in the age-18 blood and buccal DNA samples for the discordant twin pairs and the top 10 DMPs were identified using the ranked magnitude-significance method described above. The specificity of the top 10 DMPs associated with severe adolescent victimization in the longitudinal and the cross-sectional discordant twin analyses was determined by examining the within-twin DNAm differences at these loci in [1] concordant unexposed control MZ twins (both twins did not have exposure to severe adolescent victimization, group 2), and [2] concordant exposed twins (both twins exposed to severe adolescent victimization, group 3). The group differences were assessed using a one-way analysis of variance (ANOVA) and post hoc pairwise comparisons (pairwise *t* test) were performed to identify which groups were significantly different from each other.

### Differentially methylated regions analysis

We used the Python module *Comb-p* [51] to identify DMRs grouping spatially correlated DMPs (seed *P*-value<1 × 10^−4^, minimum of three probes) at a maximum distance of 500bp for both the main and exploratory analyses. DMR *P*-values were corrected for multiple testing using the Šidák correction [52] as implemented as default in *Comb-p*.

### Gene ontology pathway analysis

Illumina UCSC gene annotation was used to create a test gene list from the DMPs (*P* ≤ 5e-5) in the longitudinal and cross-sectional EWASs separately. This was performed for the main and exploratory analyses results separately. Gene ontology and pathway analysis were performed using the *missMethyl* package [53–55] which takes into account the variable number of EPIC probes associated with each gene. The KEGG pathways were also investigated using the *missMethyl* package to provide further insights into the relevant biological processes associated with the DMPs (*P* < 5e-05). Independent pathways with FDR <0.05 were considered to be associated with severe adolescent victimization.

## Results

### Longitudinal DNAm changes in MZ twins with differing exposures to adolescent victimization

**Figure 1.**
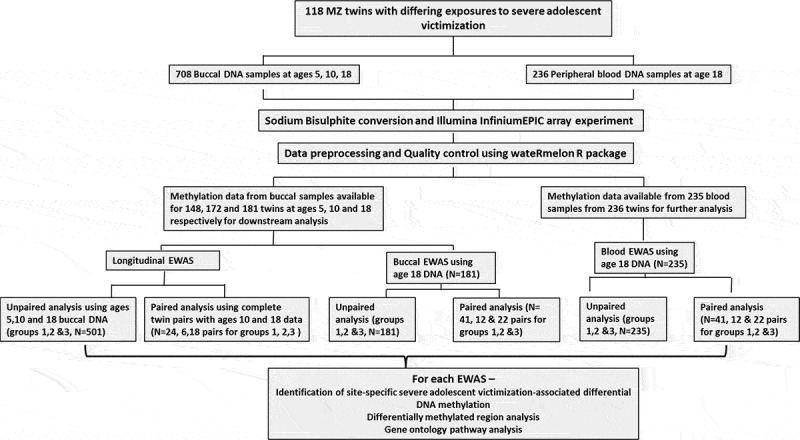
An overview of the study design

**Abbreviations**: MZ, monozygotic; EWAS, epigenome-wide association study. Group 1: discordant MZ twin pairs where only one twin in the pair had reported severe adolescent victimization; Group 2: concordant unexposed MZ twin pairs where both twins had reported no severe adolescent victimization; and Group 3: concordant exposed MZ twin pairs where both twins had reported severe adolescent victimization.

**Figure 2.**
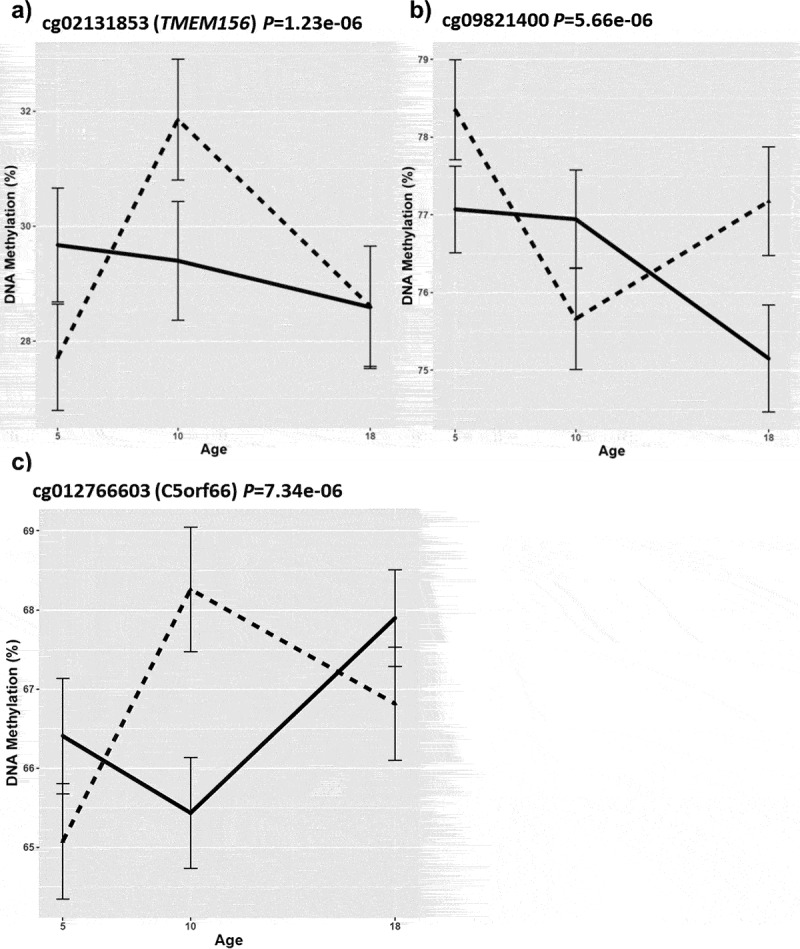
**The epigenetic trajectories for the three top ranked differentially methylated probes** (a-c) **in the longitudinal epigenome-wide association study for severe adolescent victimization in exposed (solid line) and unexposed (dotted line) twins.**

**Abbreviations**: Covariates included age, gender, cell-type proportions, and smoking pack years at age 18. The error bars represent the standard error of the mean.

An overview of our study is illustrated in Figure 1. Although none of the differentially methylated positions (DMPs) passed the stringent EPIC-array threshold (P < 9e–08) in our primary unpaired analysis, we identified 20 severe adolescent victimization-associated DMPs that passed the ‘discovery’ *P*-value threshold of *P* < 5e-5 (Table 1a, Supplementary Figure 2). The trajectories for DNAm at the three top-ranked severe adolescent victimization-associated DMPs are detailed in Figure 2a–Figure 2c. The top-ranked probe cg02131853 (∆Beta = 3.43%, *P* = 1.23e-06), mapping upstream of *TMEM156* gene, exhibited a differential trajectory of DNAm change from ages 10–18 between exposed and unexposed twins (Figure 2a).

**Table 1.** Top DMPs (*P* < 5e-05) associated with severe adolescent victimization in the unpaired analysis

a) Longitudinal EWAS using buccal samples at ages 5, 10 and 18					
**Probe**	**Genomic location (hg19)**	**Illumina gene annotation**	**Relation to UCSC CpG Island**	**DNA Methylation difference (Exp-NotExp (%))**	***P*-value**
cg02131853	Chr4:39,034,637	*TMEM156*		3.43	1.23E-06
cg09821400	Chr10:33,654,419			−3.72	5.66E-06
cg12766603	Chr5:134,493,379	*C5orf66*		4.22	7.34E-06
cg17940200	Chr2:19,170,947			−3.03	8.94E-06
cg00333899	Chr16:56,973,284	*HERPUD1*		−5.45	9.98E-06
cg01023672	Chr12:47,477,223		S_Shelf	3.44	1.44E-05
cg25886063	Chr22:23,470,899	*RSPH14*		4.12	1.53E-05
cg05622171	Chr9:80,379,662	*GNAQ*		5.33	1.76E-05
cg02932889	Chr6:144,083,663	*PHACTR2*		−5.03	1.97E-05
cg11956908	Chr2:12,694,638			−2.52	2.24E-05
cg22047262	Chr12:50,189,301	*NCKAP5L*	S_Shore	4.89	2.50E-05
cg07699901	Chr20:46,980,988			−3.32	2.61E-05
cg04159121	ChrX:23,949,463	*CXorf58*		3.76	3.17E-05
cg08296385	Chr8:94,766,346	*TMEM67*	N_Shore	5.02	3.34E-05
cg03743191	Chr10:1,258,225	*ADARB2*		2.94	4.21E-05
cg22685779	ChrX:39,548,680		Island	3.59	4.39E-05
cg19686759	Chr8:76,035,448			−3.9	4.39E-05
cg01715107	Chr14:61,069,502			−2.85	4.72E-05
cg05565668	Chr13:41,362,322	*SLC25A15*	N_Shore	3.26	4.88E-05
cg06430102	Chr19:1,151,960	*SBNO2*	N_Shore	−3.37	4.88E-05
**b) Age-18 blood EWAS**					
**Probe**	**Genomic location (hg19)**	**Illumina gene annotation**	**Relation to UCSC CpG Island**	**DNA Methylation difference (Exp-NotExp (%))**	***P*-value**
cg21566892	Chr8:68,435,064	*CPA6*		−1.58	4.16E-07
cg03508409	Chr16:30,662,237	*PRR14*	Island	−1.25	4.59E-07
cg26470696	Chr19:19,383,613	*TM6SF2*	N_Shore	−1.57	6.50E-07
cg00969565	Chr17:79,946,805	*ASPSCR1*	S_Shore	−1.16	1.84E-06
cg27614241	Chr16:50,394,527	*BRD7*		−1.51	2.76E-06
cg18721742	Chr15:72,022,092	*THSD4*		−0.9	2.87E-06
cg26295669	Chr10:56,368,156	*PCDH15*		1.95	3.11E-06
cg10505740	Chr16:2,478,800	*CCNF*	Island	0.43	3.17E-06
cg17599432	Chr5:80,443,909	*RASGRF2*		3.27	3.56E-06
cg12081027	Chr7:807,690	*DNAAF5*	Island	1.36	5.02E-06
cg21386120	Chr17:47,288,549	*GNGT2;ABI3*		−1.5	5.57E-06
cg12403329	Chr17:8,371,203	*NDEL1*		1	5.93E-06
cg23242456	ChrX:129,390,430	*ZNF280C*		1.83	7.90E-06
cg05430257	Chr12:12,218,620			2.67	7.91E-06
cg17068700	Chr4:187,877,218		N_Shelf	−1.13	9.24E-06
cg26142044	Chr5:149,325,279	*PDE6A*		2.68	9.26E-06
cg20541370	Chr6:44,549,075			−1.48	1.29E-05
cg14298020	Chr6:29,712,462	*LOC285830*		−1.47	1.36E-05
cg09634134	Chr5:321,681	*AHRR*	Island	−0.89	1.39E-05
cg08743508	Chr9:124,363,098	*DAB2IP*	S_Shore	1.46	1.45E-05
cg03982845	Chr13:26,671,869			1.03	1.45E-05
cg01919034	Chr15:69,741,819		N_Shelf	2.1	1.49E-05
cg20775917	Chr18:596,959	*CLUL1*		−0.72	1.57E-05
cg02630914	Chr20:62,436,995	*ZBTB46*	N_Shelf	−1.04	1.60E-05
cg17293936	Chr3:185,911,519	*DGKG*	Island	−1.77	1.63E-05
cg16342842	Chr10:18,270,704	*SLC39A12*		−1.52	1.68E-05
cg00879723	Chr22:29,103,292	*CHEK2*		−0.98	1.68E-05
cg25914350	Chr21:48,068,403	*PRMT2*	N_Shore	−0.92	1.70E-05
cg20036982	Chr14:31,676,999	*HECTD1*	Island	−1.06	1.75E-05
cg18194957	Chr15:74,833,314	*ARID3B*	Island	−0.85	1.94E-05
cg17808569	Chr6:168,775,292		S_Shelf	−1.11	1.96E-05
cg02754380	Chr3:186,369,639	*FETUB*		−0.89	1.98E-05
cg08361130	Chr8:91,096,340	*CALB1*		−2.17	2.19E-05
cg02540975	Chr6:153,825,208			1.17	2.21E-05
cg09324653	Chr2:135,162,250	*MGAT5*		1.53	2.35E-05
cg05396017	ChrX:37,002,530		N_Shore	1.02	2.37E-05
cg07615802	Chr2:139,660,277		Island	3.4	2.48E-05
cg10640064	Chr9:100,675,730	*TRMO*		1.5	2.49E-05
cg08274518	Chr3:33,758,023	*CLASP2*	N_Shore	0.92	2.57E-05
cg09829382	Chr19:3,286,070	*CELF5*	Island	−0.67	2.69E-05
cg08545132	Chr19:15,348,803	*BRD4*	Island	1	2.73E-05
cg08841098	Chr16:55,050,401		N_Shelf	0.85	2.87E-05
cg22069749	Chr20:62,111,366		Island	−1.31	2.90E-05
cg25150799	Chr12:52,370,254	*ACVR1B*		−0.8	2.91E-05
cg00664688	Chr6:40,544,405	*LRFN2*		−0.92	2.98E-05
cg13381110	Chr18:60,646,614	*PHLPP1*		−1.71	3.05E-05
cg14709069	Chr5:122,580,768			2.52	3.07E-05
cg07415271	Chr5:14,082,388			−1.72	3.34E-05
cg04218345	Chr3:192,232,712	*FGF12*	Island	−1.37	3.35E-05
cg01419914	Chr17:79,374,691	*BAHCC1*	Island	2.05	3.38E-05
cg10720040	Chr17:1,314,729			2.17	3.38E-05
cg04107005	Chr13:113,029,350	*SPACA7*		−1.08	3.40E-05
cg12020476	Chr7:100,183,423	*LRCH4;FBXO24*	N_Shore	−1.3	3.43E-05
cg16978268	Chr18:60,646,671	*PHLPP1*		−1.23	3.45E-05
cg05523370	Chr1:150,293,897	*PRPF3*	Island	−0.77	3.48E-05
cg07115291	Chr2:141,925,641	*LRP1B*		1.61	3.65E-05
cg07744116	Chr5:2,184,062		S_Shelf	1.32	3.78E-05
cg05672801	Chr22:38,224,528		S_Shelf	0.87	3.82E-05
cg15582138	Chr12:94,251,169			−2.02	4.04E-05
cg02783121	Chr10:85,954,092	*CDHR1*	N_Shore	0.54	4.06E-05
cg10773309	Chr1:32,229,411	*ADGRB2*	Island	−1.11	4.07E-05
cg13445575	Chr20:31,154,252	*NOL4L*		−1.16	4.09E-05
cg19624491	Chr13:109,431,045	*MYO16*		−1.51	4.12E-05
cg03516026	Chr1:16,564,651	*CPLANE2*	S_Shore	−1.4	4.25E-05
cg10611732	Chr7:37,037,291	*ELMO1-AS1;ELMO1*		1.89	4.31E-05
cg27373426	Chr1:16,330,404	*SRARP*		−1.01	4.31E-05
cg16567823	Chr16:85,815,790	*EMC8*		−1.38	4.58E-05
cg19636840	Chr16:53,393,179			−0.97	4.64E-05
cg25003598	Chr6:152,144,059	*ESR1*		1.58	4.68E-05
cg15442792	Chr6:30,951,377	*MUC21*		2.3	4.68E-05
cg17504968	Chr18:77,722,045		N_Shelf	3.04	4.81E-05
cg26831119	Chr4:111,550,830	*PITX2*	S_Shore	1.08	4.93E-05
**c) Age-18 buccal EWAS**					
**Probe**	**Genomic location (hg19)**	**Illumina gene annotation**	**Relation to UCSC CpG Island**	**DNA Methylation difference (Exp-NotExp (%))**	***P*-value**
cg20000688	Chr4:13,923,646	*LINC01182*		2.32	9.19E-07
cg17341159	Chr1:21,080,242	*HP1BP3*		−2.27	1.49E-06
cg13927700	Chr6:40,973,457	*LOC101929555*		−2.04	1.87E-06
cg06112163	Chr6:11,415,116			3.21	3.37E-06
cg19837003	Chr2:219,730,793	*WNT6*		1.72	6.15E-06
cg25037578	Chr1:247,768,540	*OR2G3*		−2.24	6.81E-06
cg11241097	Chr1:217,699,681	*GPATCH2*		−1.98	6.89E-06
cg14776738	Chr19:6,476,756	*DENND1C*	Island	−2.76	9.63E-06
cg09333325	Chr14:104,611,819	*KIF26A*		−2.98	1.06E-05
cg05089197	Chr6:11,194,815	*NEDD9*		−1.57	1.20E-05
cg22424108	Chr1:95,285,531	*SLC44A3*	N_Shore	2.16	1.48E-05
cg13131167	Chr14:32,029,920	*NUBPL*		−1.49	1.52E-05
cg10432837	Chr12:52,453,496			3.12	1.59E-05
cg04837959	Chr1:29,742,186			2.25	1.64E-05
cg05434496	Chr15:77,479,277	*PEAK1*		−2.59	1.68E-05
cg18453446	Chr21:28,219,387		S_Shore	−2.36	1.77E-05
cg18113101	Chr21:43,221,756	*PRDM15*	Island	1.83	1.84E-05
cg10493324	Chr11:118,974,627		N_Shelf	−3.6	2.01E-05
cg08379738	Chr19:6,477,033	*DENND1C*	Island	−3.13	2.40E-05
cg19425969	Chr7:157,291,474		N_Shore	−2.58	2.43E-05
cg22697786	Chr7:4,939,325			−2.15	2.56E-05
cg03428109	Chr2:207,118,288			−2.66	2.64E-05
cg21622202	Chr2:174,224,162	*CDCA7*	S_Shelf	−1.37	2.78E-05
cg09978259	Chr22:21,352,343	*LZTR1*	N_Shore	3.48	2.81E-05
cg05006942	Chr16:69,776,039	*NOB1*	N_Shore	−3.12	3.03E-05
cg08340042	Chr5:33,240,792			−1.11	3.03E-05
cg04985523	ChrX:40,149,679			1.43	3.03E-05
cg27565337	Chr13:114,856,062	*RASA3*	S_Shelf	3.01	3.06E-05
cg03754195	ChrX:119,694,964	*CUL4B*	Island	−2.97	3.07E-05
cg02883958	Chr19:18,508,710	*LRRC25*		−2.43	3.18E-05
cg10807961	Chr4:39,128,506	*MIR1273H*		−1.76	3.20E-05
cg15183258	Chr15:42,061,865	*MGA*		−3.28	3.24E-05
cg23032838	Chr14:101,394,998			−2.06	3.50E-05
cg24937727	Chr19:11,517,079	*RGL3*	Island	4.07	3.54E-05
cg02556928	Chr22:37,309,980	*CSF2RB*		3.43	3.66E-05
cg16942568	Chr1:116,561,597	*SLC22A15*		−2.85	3.98E-05
cg04880992	Chr1:67,434,664	*MIER1*		−1.81	3.99E-05
cg02452627	Chr10:31,321,325	*ZNF438*	Island	−2.16	4.05E-05
cg07421595	Chr9:34,663,026	*CCL27*	N_Shore	4.55	4.16E-05
cg20440545	Chr6:53,794,606	*LOC101927189*		2.99	4.20E-05
cg13970218	Chr7:103,965,666		N_Shelf	−1.3	4.59E-05
cg11305999	Chr12:66,449,691			−1.91	4.66E-05

*Covariates included age, gender, smoking pack years, and cell-type proportions. Chr, chromosome; DMP, differentially methylated probe; EWAS, epigenome-wide association study; Exp, exposed to any severe victimization during adolescence; NotExp, not exposed to any severe victimization during adolescence; UCSC, University of California Santa Cruz.*

In our exploratory paired analysis, where we investigated the within twin-pair longitudinal ∆β change between ages 10 and 18 in the discordant MZ twin pairs (n = 24) using the ranked magnitude-significance method, we identified the cg09348925 probe as the most associated finding (∆Beta = 15.4%, *P* = 1.32e-05), located on chromosome 20 with the closest gene being a zinc finger protein gene *ZNF217* (approx. 200 kb upstream) (Table 2a, Supplementary Figure 3a). We next tested the specificity of the top 10 severe adolescent victimization-associated DMPs by comparing the average within-twin longitudinal DNA methylation differences with those from six age-matched concordant unexposed MZ twin pairs (where neither twin was exposed to severe adolescent victimization) and 18 concordant exposed MZ twin pairs (where both the twins were exposed to severe adolescent victimization). At two of the top-10 DMPs, the average within-twin ∆β was significantly larger in the discordant twins compared to the concordant exposed and concordant unexposed twins (see Supplementary Figure 3b) with post hoc pairwise comparisons indicating that average within-twin differences in DNA methylation are significantly larger at these top-ranked DMPs in the discordant twins compared to the twins concordant for exposure to severe adolescent victimization.

**Table 2.** Top ranked list of DMPs associated with severe adolescent victimization in the paired analysis of discordant MZ twin pairs

a) Longitudinal EWAS using buccal samples at ages 10 and 18							
**Probe**	**Exposed twin longitudinal ∆β mean**	**Unexposed co-twin longitudinal ∆β mean**	**Mean longitudinal ∆β difference**	***P*-value**	**Genomic location (hg19)**	**Illumina gene annotation**	**Relation to UCSC CpG_Island**
cg09348925	0.101	−0.053	0.154	1.32E-05	Chr20:52,422,424		
cg17121416	−0.089	0.048	−0.137	5.30E-06	Chr13:24,024,605		
cg18434560	0.081	−0.049	0.129	8.17E-08	Chr11:317,767		N_Shore
cg16106624	0.094	−0.041	0.135	2.90E-05	Chr12:22,852,788		
cg08643128	0.066	−0.057	0.123	1.10E-05	Chr19:39,432,669	*FBXO17*	
cg24594818	−0.111	0.027	−0.138	9.72E-05	Chr1:35,525,367	*ZMYM1*	
cg11034672	−0.109	0.034	−0.143	1.18E-04	Chr14:75,151,321	*AREL1*	
cg24854698	0.114	−0.006	0.12	6.37E-06	Chr5:99,875,480	*FAM174A*	S_Shelf
cg12662887	−0.082	0.069	−0.15	1.45E-04	Chr10:105,343,920	*NEURL1*	N_Shore
cg18115721	−0.046	0.081	−0.127	1.01E-04	Chr11:73,567,838	*MRPL48*	
**b) Age-18 blood EWAS**							
**Probe**	**Exposed twin mean**	**Unexposed co-twin mean**	**Mean ∆β**	***P*-value**	**Genomic location (hg19)**	**Illumina gene annotation**	**Relation to UCSC CpG_Island**
cg25412677	0.741	0.709	0.033	4.80E-05	Chr3:181,661,470		
cg08884029	0.742	0.709	0.034	1.56E-04	Chr12:110,012,500	*MVK;MMAB*	S_Shore
cg09784461	0.677	0.642	0.035	2.29E-04	Chr15:70,962,357	*UACA*	
cg13202527	0.675	0.707	−0.032	1.67E-04	Chr2:219,232,091	*CATIP;CATIP-AS1*	N_Shore
cg09152353	0.739	0.706	0.033	2.76E-04	Chr2:10,134,152	*GRHL1*	
cg27380880	0.575	0.608	−0.032	2.23E-04	Chr12:65,022,110	*RASSF3*	
cg26821000	0.674	0.641	0.032	2.88E-04	Chr2:773,393		
cg13069411	0.71	0.676	0.034	3.88E-04	Chr11:65,771,070	*EIF1AD;BANF1*	S_Shore
cg00854925	0.391	0.427	−0.036	5.16E-04	ChrX:152,689,318		S_Shelf
cg25593022	0.799	0.768	0.03	5.63E-05	Chr3:190,294,628	*IL1RAP*	
**c) Age-18 buccal EWAS**							
**Probe**	**Exposed twin mean**	**Unexposed co-twin mean**	**Mean ∆β**	***P*-value**	**Genomic location (hg19)**	**Illumina gene annotation**	**Relation to UCSC CpG_Island**
cg12971523	0.529	0.595	−0.066	3.60E-05	Chr6:170,479,382		S_Shelf
cg24937727	0.195	0.136	0.059	3.90E-05	Chr19:11,517,079	*RGL3*	Island
cg04245104	0.603	0.668	−0.065	1.90E-04	Chr1:115,873,370	*NGF*	
cg15440099	0.369	0.313	0.056	7.10E-05	Chr3:44,293,944	*TOPAZ1*	
cg05850732	0.557	0.503	0.054	9.60E-05	Chr2:225,882,503	*DOCK10*	
cg18655494	0.55	0.605	−0.055	1.50E-04	Chr20:14,542,753	*MACROD2-IT1;MACROD2*	
cg12662887	0.55	0.629	−0.079	3.50E-04	Chr10:105,343,920	*NEURL*	N_Shore
cg02061711	0.688	0.744	−0.056	2.50E-04	Chr10:601,759	*DIP2C*	
cg20118157	0.65	0.711	−0.061	5.40E-04	Chr3:193,078,051	*ATP13A5*	
cg12811011	0.418	0.365	0.054	4.10E-04	Chr15:69,827,705		

**Notes**: Probes ranked by a combination of both mean absolute difference in methylation level (**∆β**) and statistical significance. Chr, chromosome; DMP, differentially methylated probe; EWAS, epigenome-wide association study; hg19, Human Genome version 19; MZ, monozygotic; UCSC, University of California Santa Cruz.

No differentially methylated regions (DMRs) were identified in our longitudinal unpaired and paired analyses. Downstream gene ontology (GO) and KEGG enrichment analysis on genes annotated to the severe adolescent victimization-associated DMPs (*P* < 5e-05) in the unpaired analyses identified significant enrichment of associated DMPs in KEGG pathways including lipid metabolism and inflammatory mediator regulation of TRP channels (Supplementary Table 2). In our KEGG analysis of the paired longitudinal EWAS results, 16 pathways were significantly associated (FDR<0.5) with severe adolescent victimization including linoleic acid metabolism and arachidonic acid metabolism pathways (Supplementary Table 3) common to the unpaired KEGG pathway results.

### Site-specific DNAm differences in MZ twins with differing exposures to severe adolescent victimization in blood and buccal DNA at age 18

In our unpaired cross-sectional age-18 blood EWAS, we observed considerable variability in the DNAm at individual CpG sites within severe adolescent victimization-exposed and unexposed twins, although none of the DMPs survived multiple testing, in line with those reported by Marzi *et al*.[14] in a related analysis of the full E-Risk cohort. Specifically, our age-18 blood dataset revealed 72 severe victimization-associated DMPs (*P* < 5e-5; Table 1b, Supplementary Figure 4) annotated to 54 genes with effect sizes (mean methylation difference between exposed and unexposed groups) ranging from −2.2% to 3.4% (Table 1b). The top ranked DMP cg21566892, which mapped to the intragenic region of the *CPA6* gene encoding a metallocarboxypeptidase, was significantly hypomethylated (∆Beta = −1.6%, *P* = 4.16e-07) in severe victimization-exposed twins compared to unexposed twins (Supplementary Figure 5a).

In the exploratory paired age-18 blood analysis including DNAm data from 41 discordant twin pairs (complete pairs with age-18 blood and buccal data after QC), we identified the cg25412677 probe on Chr3q26.33 as the most associated finding (Beta = ∆3.3%, *P* = 4.80e-05) (Table 2b, Supplementary Figure 6a). Of note, we did not observe any significant difference in smoking behavior between the exposed and unexposed twins within the twin pairs group discordant for severe adolescent victimization exposure (*P* = 0.573). Interestingly, we reported significant differences in the average within-twin methylation values for seven of the top 10 associated loci between the discordant twins and concordant exposed and unexposed twins (groups 1, 2 and 3, Supplementary Figure 6b), with three of these showing higher average within-twin methylation differences in the discordant twin group compared to both the concordant exposed and unexposed twins (Supplementary Figure 6b).

**Figure 3.**
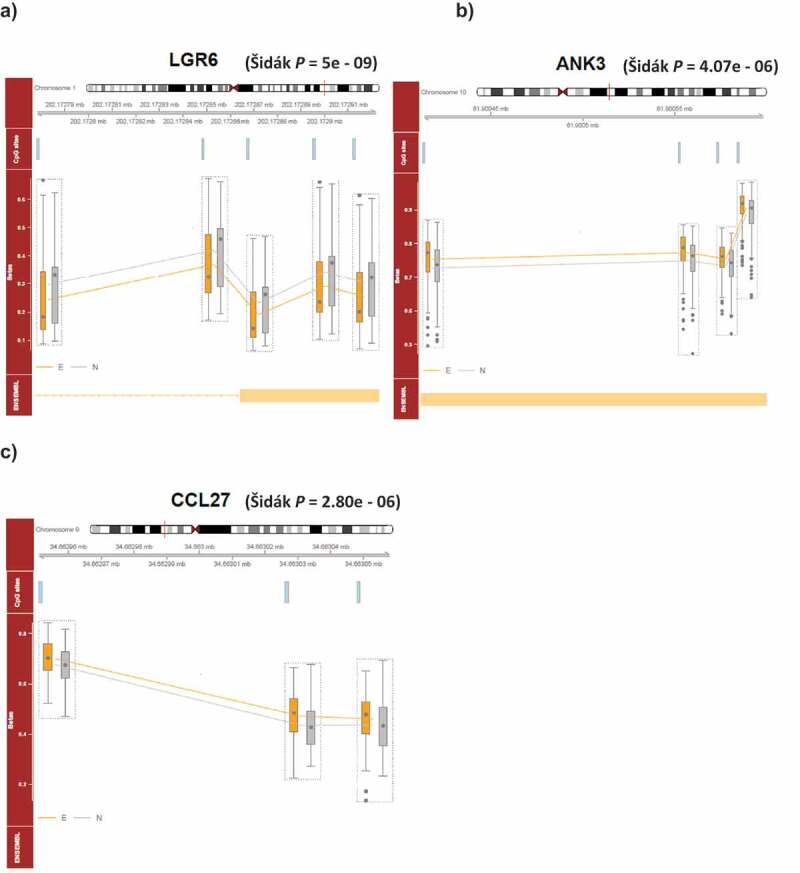
**DNA methylation profiles of probes identified within the severe adolescent victimization-associated DMRs, including** (a) ***LGR6* and** (b) ***ANK3* in the age-18 blood, and c) *CCL27* in the age-18 buccal epigenome-wide association study.**

**Abbreviations**: Covariates included gender, smoking pack years at age 18, and cell-type proportions. E, twins exposed to any severe adolescent victimization, N = twins not exposed to any severe adolescent victimization, DMRs = differentially methylated regions.

Using a regional approach, we identified two victimization-associated DMRs in *LGR6* (Šidák *P*-value: *P* = 5e-09) and *ANK3* (Šidák *P*-value: *P* = 4.07e-06) (Figures 3a and 3b, Supplementary Table 4) in the unpaired EWAS. No DMRs were identified in our paired discordant twin analysis. Downstream pathway analysis of the unpaired and paired age-18 blood EWAS results did not reveal any enrichment of independent GO and KEGG pathways.

To check for the robustness of the age-18 blood EPIC results, we performed additional exploratory analyses on overlapping 450 K data on matched samples and observed some evidence of consistency in the directional effect of 33 out of 72 (*P* < 5e-05) severe adolescent victimization-associated probes common across the EPIC and 450 K arrays (*P_binomial_ *= 0.01) (*Supplementary Figure 7*). However, it is worth noting that the individual probe correlations for the DNAm value across the two arrays were variable including that for probes reported in the current study with *P* < 5e-05 (21% sites with *r* > 0.5) (as detailed in *Supplementary Table 5*).

In the age-18 buccal unpaired EWAS, we identified 42 DMPs (mapped to 28 genes, *P* < 5e-5) with effect sizes ranging from −3.6% to 4.55% (Table 1c, Supplementary Figure 8) that were associated with severe adolescent victimization. The top-ranked DMP, cg20000688 (Beta = 2.32%, *P* = 9.19e-07, *Supplementary Figure 5b*), was hypermethylated in twins exposed to severe adolescent victimization compared to the unexposed twins and mapped to a long noncoding RNA gene *LINC01182*. In the exploratory paired discordant twin analysis, the probe cg12971523 (∆Beta = −6.6%, *P* = 3.6e-05, Table 2c, Supplementary Figure 9a) was the most associated finding. Of note, the second ranked probe cg24937727 (∆Beta = 5.9%, *P* = 3.9e-05) was also associated (*P* < 5e-05) in the unpaired analysis (∆Beta = 4.07%, *P* = 3.54e-05, Table 1c) and is located intragenic in a CpG island in *RGL3* suggesting a robust methylation difference in the age-18 buccal tissue of the exposed and unexposed twins. At five of the 10 top-ranked DMPs, the average within-twin differences in DNA methylation were significantly different between the groups (Supplementary Figure 9b).

We identified a DMR associated with severe victimization upstream of the *CCL27* gene (Šidák *P*-value: *P* = 2.80e-06, Figure 3c, Supplementary Table 4) in the age-18 buccal unpaired regional analysis. Pathway analysis did not identify any enrichment of GO and KEGG biological pathways in the unpaired analysis, however, for the paired age-18 buccal EWAS results, the GO analysis revealed homophilic cell adhesion via plasma membrane adhesion molecules pathway (*Supplementary Table 6*).

### Severe adolescent victimization-associated methylomic differences are shared between peripheral tissues

**Figure 4.**
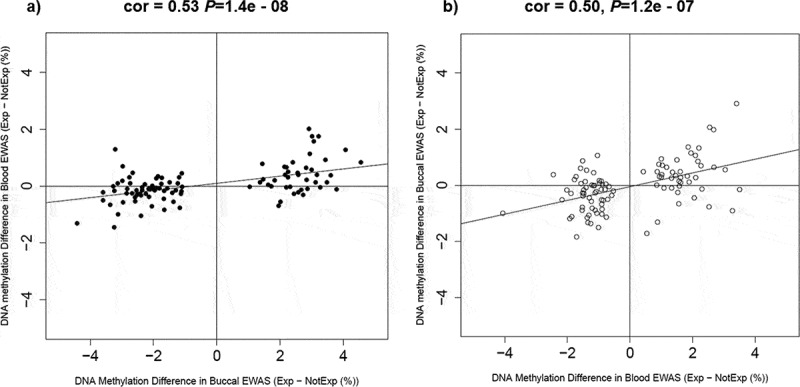
**The effect sizes of the top 100 severe adolescent victimization-associated DMPs in** (a) **age-18 buccal, and** (b) **age-18 blood samples showed strong significant positive correlations with the severe adolescent victimization-associated effect sizes of the same probes from the other peripheral tissue type.**

**Abbreviations**: Exp = twins exposed to any severe adolescent victimization, NotExp = twins not exposed to any severe adolescent victimization. DMPs = differentially methylated probes.

We next examined the extent to which severe adolescent victimization-associated DNAm differences are shared between tissue types (blood and buccal) using results from the unpaired analysis. Despite the distinct lists of the top 100 adolescent victimization-associated DMPs in the age-18 blood and age-18 buccal EWAS, there were positive correlations between the effect sizes of victimization-associated DMPs in the two datasets. Specifically, the effect sizes of the top 100 severe victimization-associated DMPs (i.e., the change in DNA methylation at the probe-level as a result of victimization, controlling for cell composition, smoking history and gender) in the buccal EWAS were moderately positively correlated with those of the same probes in the blood dataset (*r* = 0.53, *P* = 1.4e-08, Figure 4a). Similarly, moderate cross-tissue positive correlations were present for the effect sizes of the top 100 DMPs in the blood EWAS analyses when compared to the buccal dataset (*r* = 0.50, *P* = 1.2e-07, Figure 4b).

## Discussion

To our knowledge, this represents the first comprehensive analysis of DNAm in relation to severe adolescent victimization that utilized a combined longitudinal, MZ twin discordance and genome-wide approach, also interrogating the potential tissue-specific epigenetic signatures associated with severe adolescent victimization using DNA collected from the same individual. In this study we performed an EWAS of severe adolescent victimization using two different statistical approaches, i.e. unpaired and paired linear regression (the former including clustered robust standard errors to account for the non-independence of twin observations). The unpaired method allowed us to correct for the effects of smoking, cell-types and gender on individual DNA methylation levels along with the inclusion of methylation data from all individuals thereby maximizing the power in detecting differential methylation associated with victimization exposure. The exploratory paired analyses explored the within twin-pair differences using the MZ twins discordant for severe adolescent victimization exposure, allowing us to control for genetic and unmeasured shared environmental influences, and we then investigated the specificity of the associated loci in concordant victimization-exposed and concordant unexposed twin pairs.

We report nominally-significant (*P* < 5e-5) altered epigenetic longitudinal trajectories associated with severe adolescent victimization at numerous CpG sites from our unpaired analysis, in genomic regions associated with stress response pathways including *TMEM67* and *HERPUD1* [56,57]. Our parallel cross-sectional unpaired EWASs in blood and buccal samples obtained from the same individuals at age 18 reported a non-overlapping list of nominally-significant (*P* < 5e-5) severe adolescent victimization-associated DMPs with some evidence of convergent signals between these two peripheral tissue types. Our exploratory paired analyses identified a DMP, cg12662887, common to the longitudinal and cross-sectional analyses. Future replication of this locus is required in independent samples. Notably, a CpG site in *RGL3* was found to be significantly differentially methylated in the age-18 buccal samples in both our paired and unpaired analyses, suggesting a robust methylation change at this locus as a result of exposure to severe adolescent victimization. Interestingly, a DMR containing this CpG (Chr 19: 11,517,079–11,517,436) has previously been associated with alcohol intake in leukocyte DNA from women participating in the European Prospective Investigation into Cancer and Nutrition (EPIC) study [58]. Another study investigating the effect of prenatal alcohol exposure on DNAm in buccal DNA from children with foetal alcohol spectrum disorder reported association with the same DMR [59]. Given that victimized adolescents are more likely to consume alcohol [60], it is plausible that our observed methylation change in *RGL3* may be a reflection of this and future studies should further explore this link.

Although there has been a recent expansion in the literature documenting DNAm variation with early-life adversity specifically in childhood [13,20,61], there is a paucity of studies in this field focusing on victimization during adolescence, with a bigger gap in the area of longitudinal research. A recent cross-sectional EWAS using whole blood in the complete E-Risk cohort of MZ and DZ twins reported limited evidence for an association between DNAm and several forms of early-life victimization [14]. We did not identify any Bonferroni-corrected significant associations between DNAm changes and severe adolescent victimization in our age-18 blood EWAS mirroring the results from the previous study, however, there are also fundamental differences between these studies. Firstly, our use of the EPIC array compared to the 450 K array increased the coverage of CpG sites across the genome nearly two-fold, and secondly, only individuals free of any severe childhood victimization but who experienced severe adolescent victimization were included in the current study, and we focused only on MZ twins. Nevertheless, we examined methylation data available for the overlapping probes on the EPIC and 450 K arrays in matched samples from the two studies (see Supplementary Table 5). Despite observing some consistency in the direction of the associations between victimization and DNAm in age-18 blood when using the 450 K and the EPIC arrays (Supplementary Figure 7), the degree of overlap appears inconclusive and thus does not provide strong support for this part of the findings. The overall correlation between the two arrays was high (r = 0.99), although individual site correlations were variable including that for probes reported in the current study with *P* < 5e-05 (21% sites with r > 0.5), which is very similar to that reported in previous studies comparing the two arrays [62,63]. Therefore, this aspect of our results should be interpreted with caution and future studies using only data generated from one type of array might have limited replicability and generalizability.

Our regional analysis (unpaired approach) identified DMRs upstream of *CCL27* in the age-18 buccal EWAS and in the genes *LGR6* and *ANK3* in the age-18 blood EWAS. The 135bp DMR in *ANK3* was consistently hypermethylated in twins exposed to severe adolescent victimization. ANK3 is a scaffolding protein and genetic variants annotated to this gene are associated with various psychiatric disorders including schizophrenia, bipolar disorder, and autism [64–66]. A recent cross-species combined methylome analysis performed in different tissues and time-points in rats, non-human primates and humans, all characterized by early-life stress, revealed consistent hypermethylation in *ANK3* in the stressed groups across all the conditions and species [67]. Findings from our study provide further support for a potentially important role of altered *ANK3* DNAm in relation to stress exposure. It is interesting to note that some of the genes identified in our differential methylation analysis are expressed in both salivary glands and leukocytes, including *RGL3, ANK3* and *CCL27*, raising the possibility of gum infection in our twins, despite adjusting for cell-type composition in our main analyses. Future studies that contain adolescent victimization exposure and dental records could explore this potentially interesting association further.

This study has several strengths. Firstly, our longitudinal design allowed us to ascertain the longitudinal epigenetic trajectories associated with severe adolescent victimization by comparing the DNA methylome of individuals before and after the exposure experience. Secondly, our MZ twin design allowed us to ascertain a purer effect of adolescent victimization on the epigenome controlling for potentially important confounders in epigenetic studies such as genetic variation, age, gender, and shared environmental exposure effects. Thirdly, known important confounders in epigenetic studies including smoking [68] and cell type composition in blood and buccal cells [69,70] were controlled for in our analyses, thereby, disentangling their mediating effects on DNAm. Methylation at *AHRR*, especially at cg05575921, in blood has been consistently reported to be inversely associated with cigarette smoking [71,72]. In the current study, one of the severe adolescent victimization-associated DMPs in age-18 blood data was located in the first intron of *AHRR* (cg09634134, ∆Beta = −0.89%, *P* = 1.39E-05), highlighting the potential intricate relationship between smoking behaviours and severe stress exposures as victimized adolescents are more likely to smoke [14,73]. It is also possible that unmeasured confounders such as smoking intensity, duration, passive smoking or air pollution [74] could explain some of the effects at this locus. We used pack years information to control for smoking for consistency in all our blood and buccal analyses rather than a smoking score, the latter being derived from DNA methylation values of smoking-associated CpG sites measured in blood samples (and thus not necessarily applicable to buccal samples). Lastly, our study sample came from a cohort that represents the full range of socioeconomic conditions in Great Britain and had 93% retention over 18 years, thereby, minimizing ascertainment and attrition bias.

Nonetheless, the results from our study should be considered in light of certain limitations. We were unable to explore associations between the DNA methylome and specific types of victimization due to the modest sample size of each subgroup. It is possible that subtype-specific analyses would yield further insights given recent findings of associations between altered DNA methylome and exposure to sexual and physical abuse [75,76]. Also, DNAm was quantified using the Illumina EPIC array; although this is a robust, highly reliable, and currently the best high-throughput platform with content spanning regulatory regions associated with the majority of known annotated genes, it interrogates DNAm at a relatively small proportion of sites across the whole genome. In this study, genome-wide DNAm profiling was performed on DNA extracted from buccal cells collected at ages 5, 10 and 18 and whole blood collected at age 18. There is no archived collection of longitudinal brain samples from twins discordant for severe adolescent victimization and no techniques currently available to explore DNAm in the brains of live individuals. Finally, there is increasing awareness of the importance of 5-hydroxymethyl cytosine (5-hmC) as an epigenetic marker [77], although this modification cannot be distinguished from DNAm using standard bisulphite-based approaches. It is plausible that many of the adolescent victimization-associated differences identified in this study are confounded by modifications other than DNAm, however, it is important to note that markers such as 5-hmC are known to be highly expressed in brain tissues but at a much lower level in blood cells.

In summary, this is the first systematic longitudinal MZ twin discordant study to examine the association between genome-wide DNAm in severe adolescent victimization, providing preliminary evidence for altered DNA methylomic signatures in individuals exposed to severe victimization during adolescence, a key stage of development and a crucial period for the onset of psychiatric disorders. Follow-up studies are needed to explicitly test whether the severe adolescent victimization-associated DMPs identified in the present study associate with psychopathological outcomes and, if so, whether they may mediate the influence of severe adolescent victimization on later mental health.

## Supplementary Material

Supplemental Material
