# Supplementary material for: DNA methylation signatures of adolescent victimization: analysis of a longitudinal monozygotic twin sample
Source: Epigenetics. 2020 Dec 29;16(11):1169–86. doi: 10.1080/15592294.2020.1853317 (PMC8813077; doi:10.1080/15592294.2020.1853317)
Supplement: Supplemental Material [file KEPI_A_1853317_SM3941.zip › Supplementary Figures_revised_final.docx]

**SUPPLEMENTARY FIGURES**

**
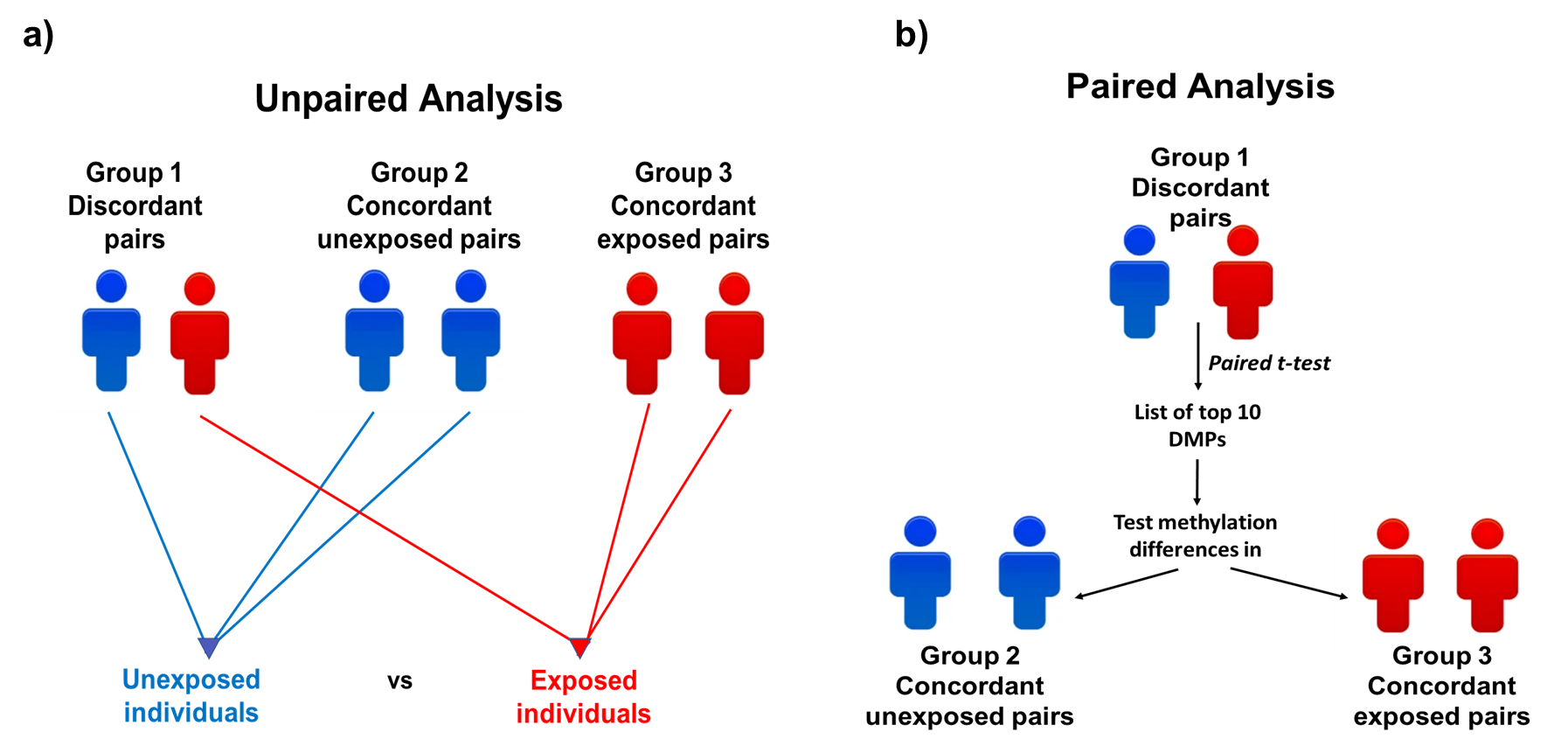
**

**Supplementary Figure 1. Schematic description of the statistical analyses, specifically a) unpaired and b) paired analysis, in this study.** *Note. Exposed, exposure to any severe adolescent victimization, DMP, differentially methylated probes.*

**2a)**

**
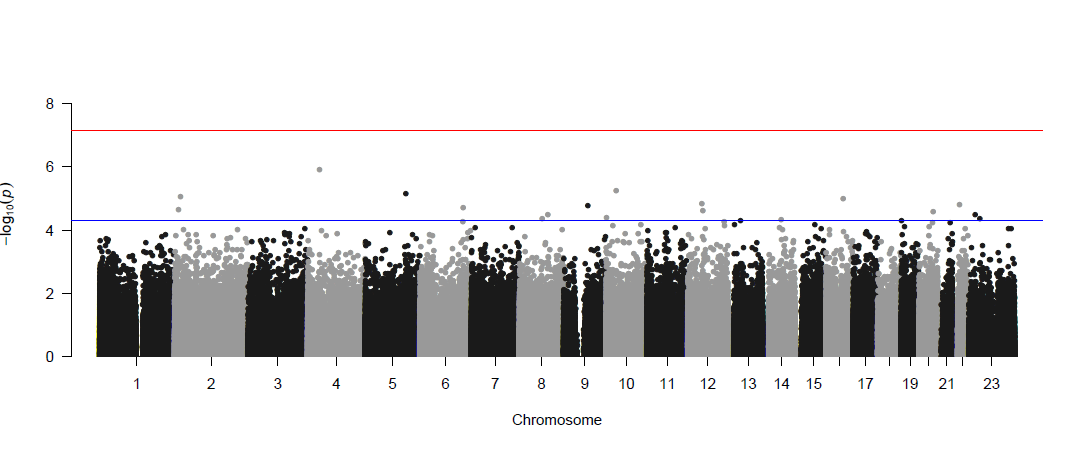
**

**2b)**

**
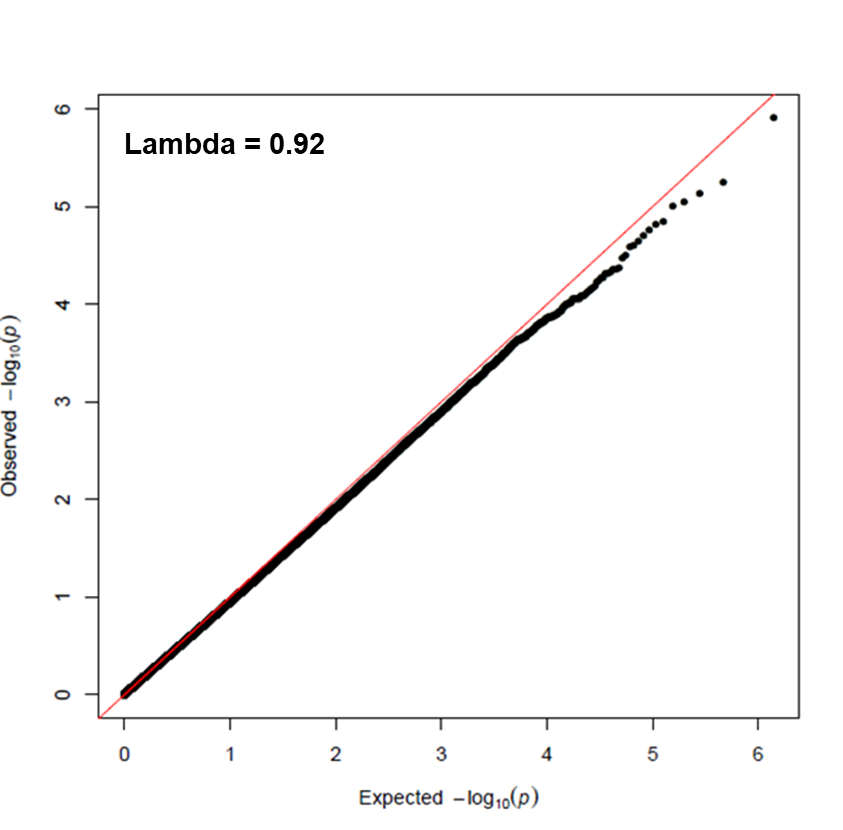
**

**Supplementary Figure 2. (a) Manhattan plot and (b) QQplot showing *P*-values from a linear regression with cluster-robust standard errors used to identify differential methylation in buccal samples associated with severe adolescent victimization exposure in the unpaired longitudinal epigenome-wide association study (red line – EPIC Array *P*-value threshold of 9e-08 and blue line – Discovery *P*-value threshold of 5e-05).** The inflation factors λ was 0.92, indicating minimal evidence for genomic deflation/inflation. *Note. Covariates included age, gender, cell-type proportions and smoking pack years.*

**Supplementary Figure 3a. Graphs showing the difference in longitudinal DNA methylation (∆β) from age 10 to age 18 between each of the 24 pair of monozygotic (MZ) twins discordant for adolescent victimization (exposed twin – unexposed co-twin) for each of the 10 top-ranked probes using the ranked magnitude-significance method. Mean within-twin pair ∆β across all 24 MZ twin-pairs is also presented.** Consistent within-twin pair differences in DNA methylation at age 18 are observed across the majority of discordant MZ twin pairs (24 pairs) at the 10 top-ranked differentially methylated positions (DMPs).

**
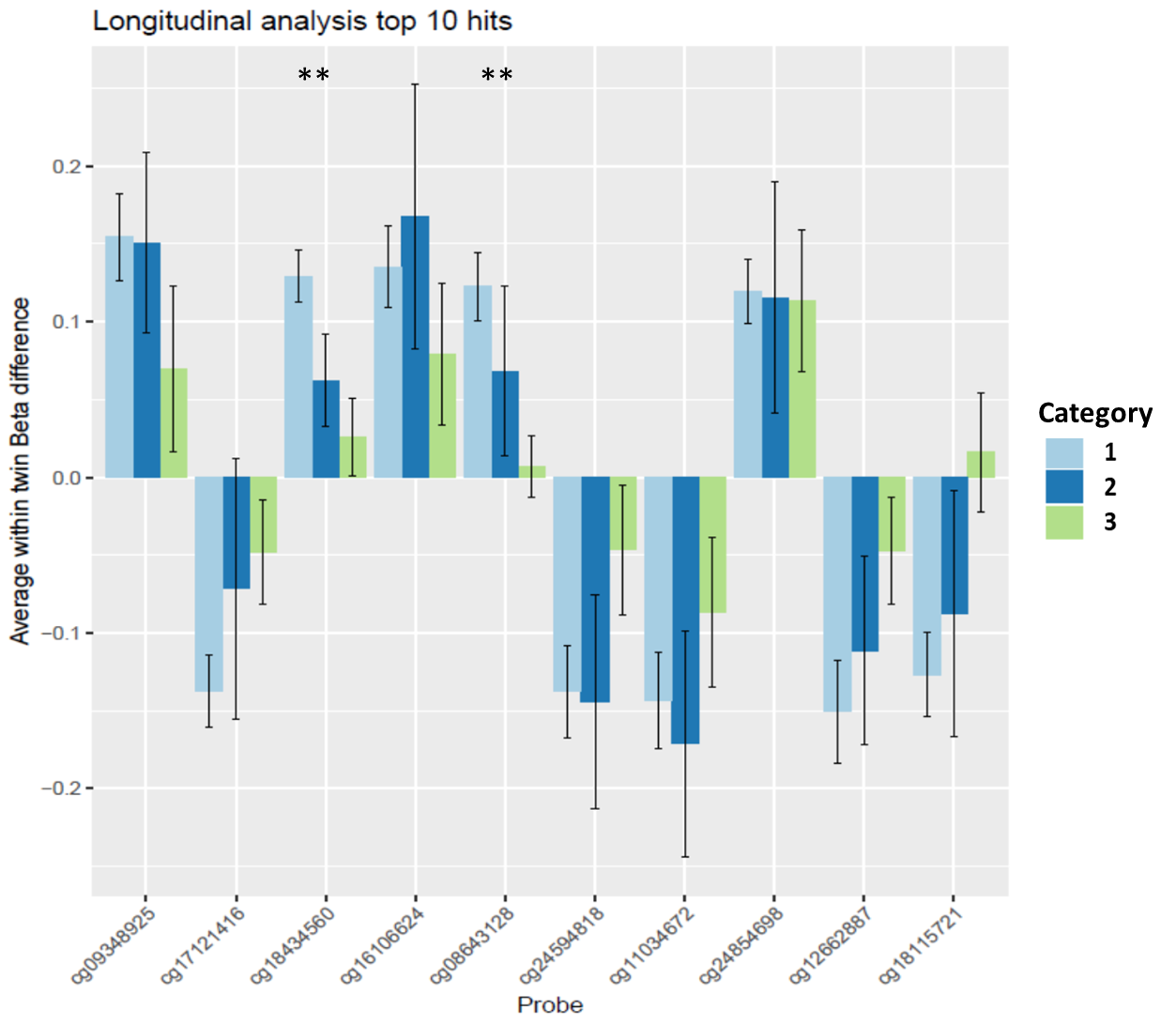
**

**Supplementary Figure 3b. Graph showing average within-twin intra-individual β difference (long ∆β = mean(age-18 beta – age-10 beta)) in buccal DNA of adolescent victimization exposure-discordant monozygotic (MZ) twins (Group 1 = 24 pairs) and the age-matched concordant unexposed MZ twins (Group 2 = 6 pairs) and concordant age-matched exposed MZ twins (Group 3 = 18 pairs).** *Note. Error bars represent +/- the standard error of the mean within-twin intra-individual ∆β. ****P < 0.0001, ***P < 0.001, **P < 0.01, *P < 0.05*

**4a)
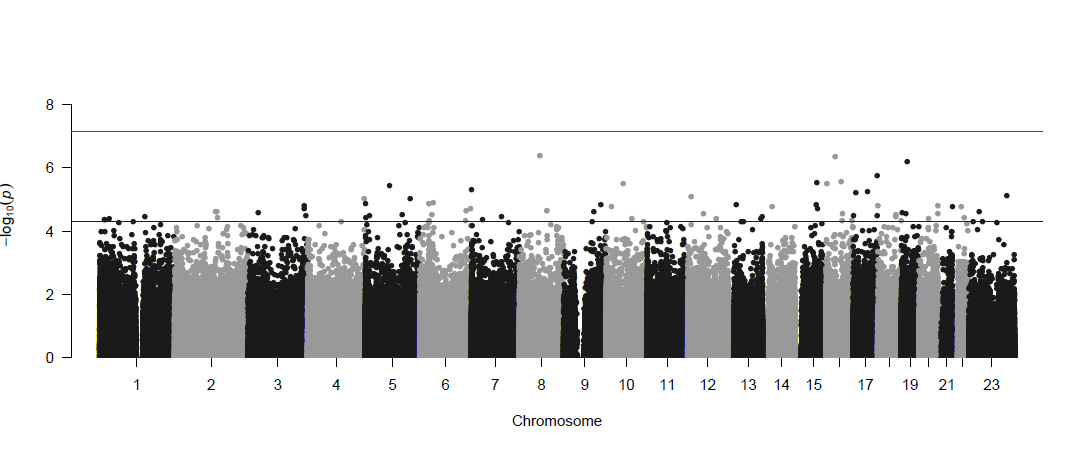
**

**4b)**

**
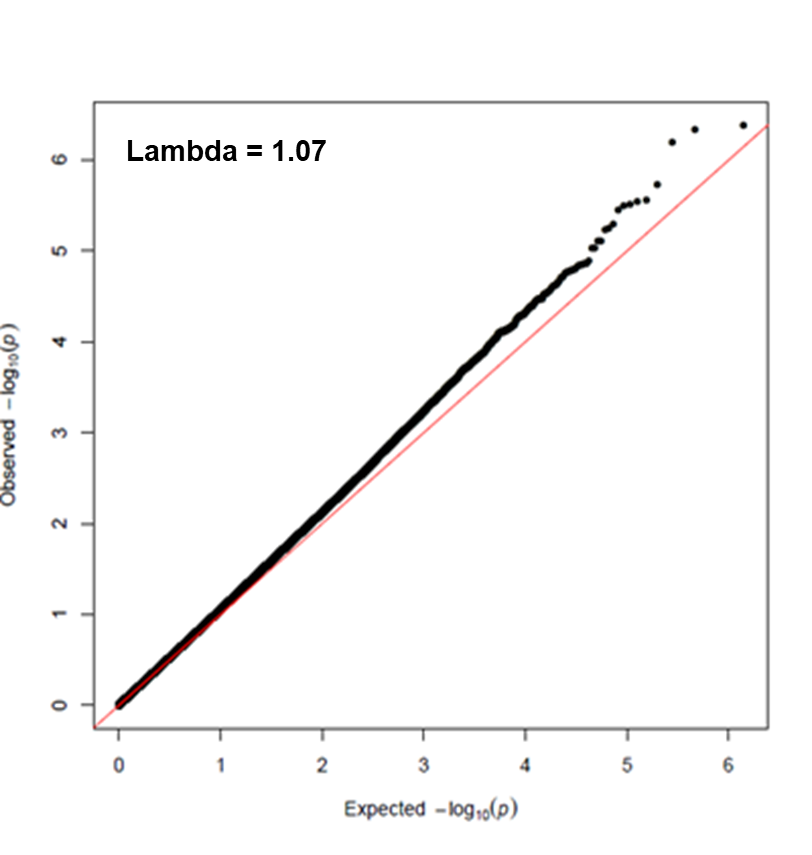
**

**Supplementary Figure 4. (a) Manhattan plot and (b) QQplot showing *P*-values from a linear regression with cluster-robust standard errors used to identify differential methylation associated with any severe adolescent victimization exposure in the age-18 blood epigenome-wide association study (red line – EPIC array *P*-value threshold of 9e-08 and blue line - Discovery *P*-value threshold of 5e-05).** The inflation factors λ was 1.07, indicating minimal evidence for genomic deflation/inflation. *Note. Covariates included gender, smoking pack years, and cell type proportions***.**

**5a) 5b)**


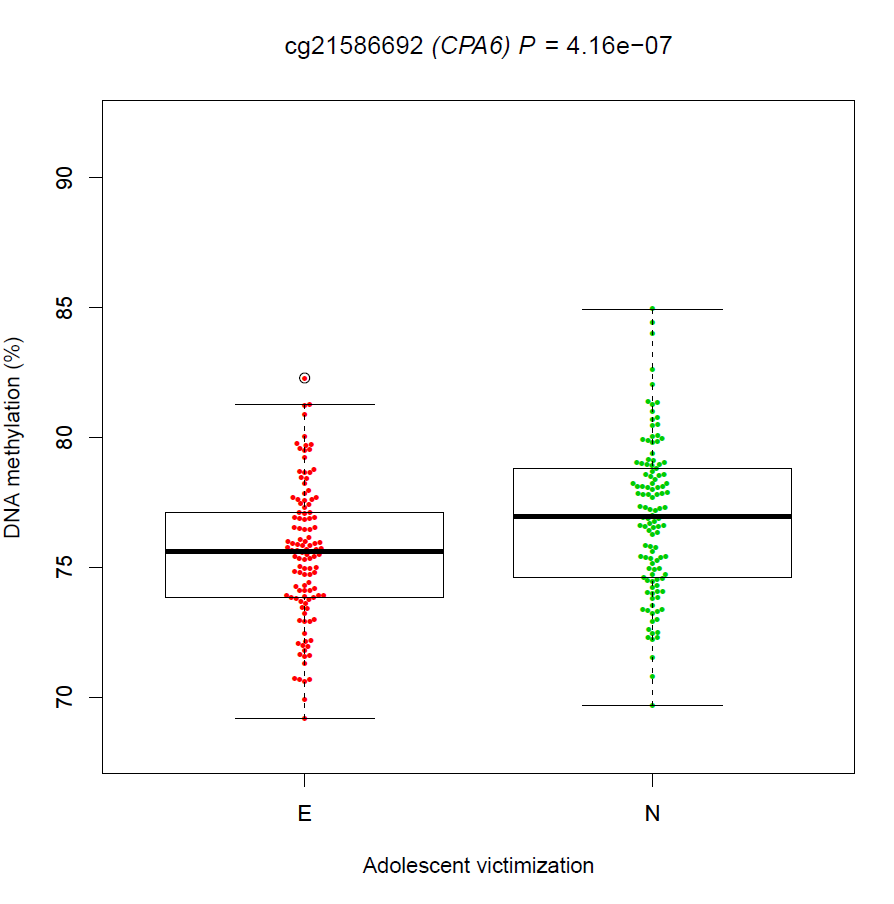

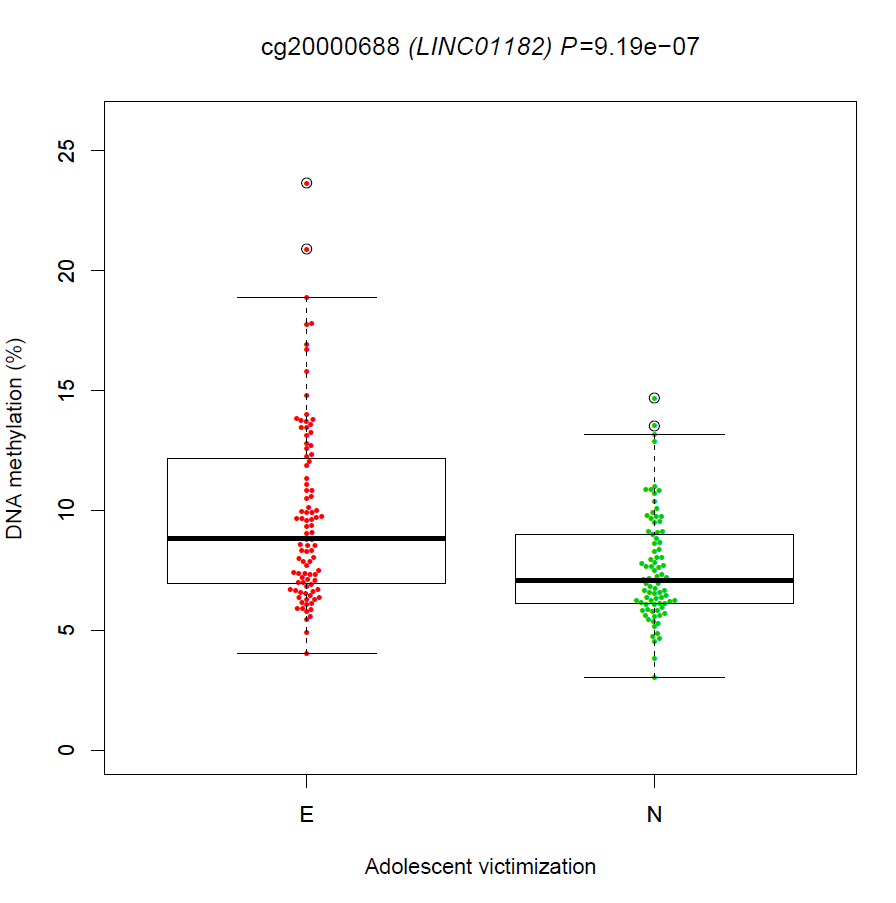


**Supplementary Figure 5. Beeswarm plots showing the DNA methylation values (%) for the top-ranked differentially methylated probes associated with severe victimization during adolescence in (a) age-18 blood, and (b) age-18 buccal epigenome-wide association studies.** *Note.* *E=twins exposed to any severe adolescent victimization, N=twins not exposed to any severe adolescent victimization.*

*
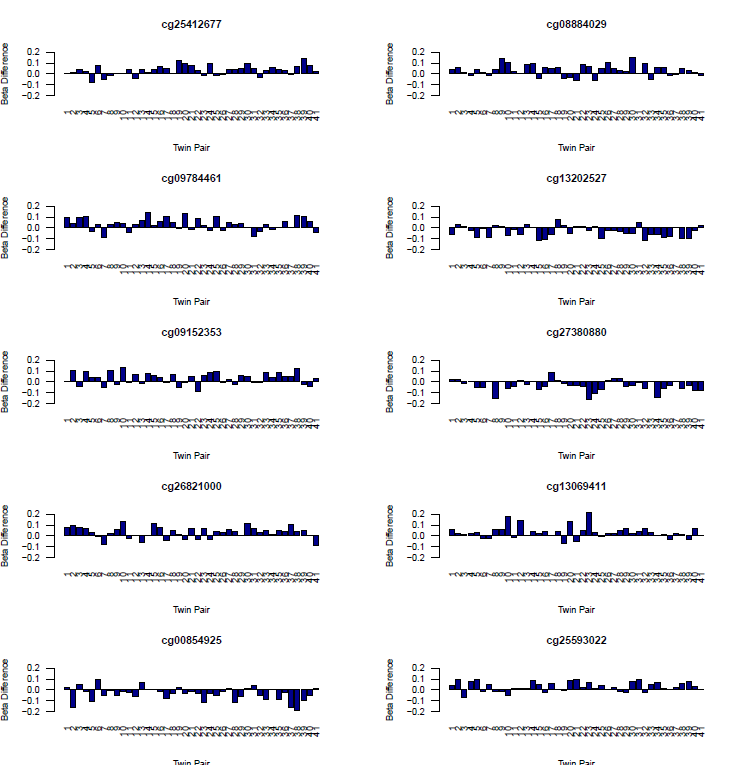
*

**Supplementary Figure 6a. Graphs showing the difference in DNA methylation (∆β) at age 18 between each pair of monozygotic (MZ) twins discordant for adolescent victimization at age 18 (exposed twin – unexposed co-twin) for each of the 10 top-ranked probes in blood samples.** Consistent within-twin pair differences in DNA methylation at age 18 are observed across discordant MZ twin pairs (41 pairs) at the 10 top-ranked differentially methylated positions (DMPs).

**
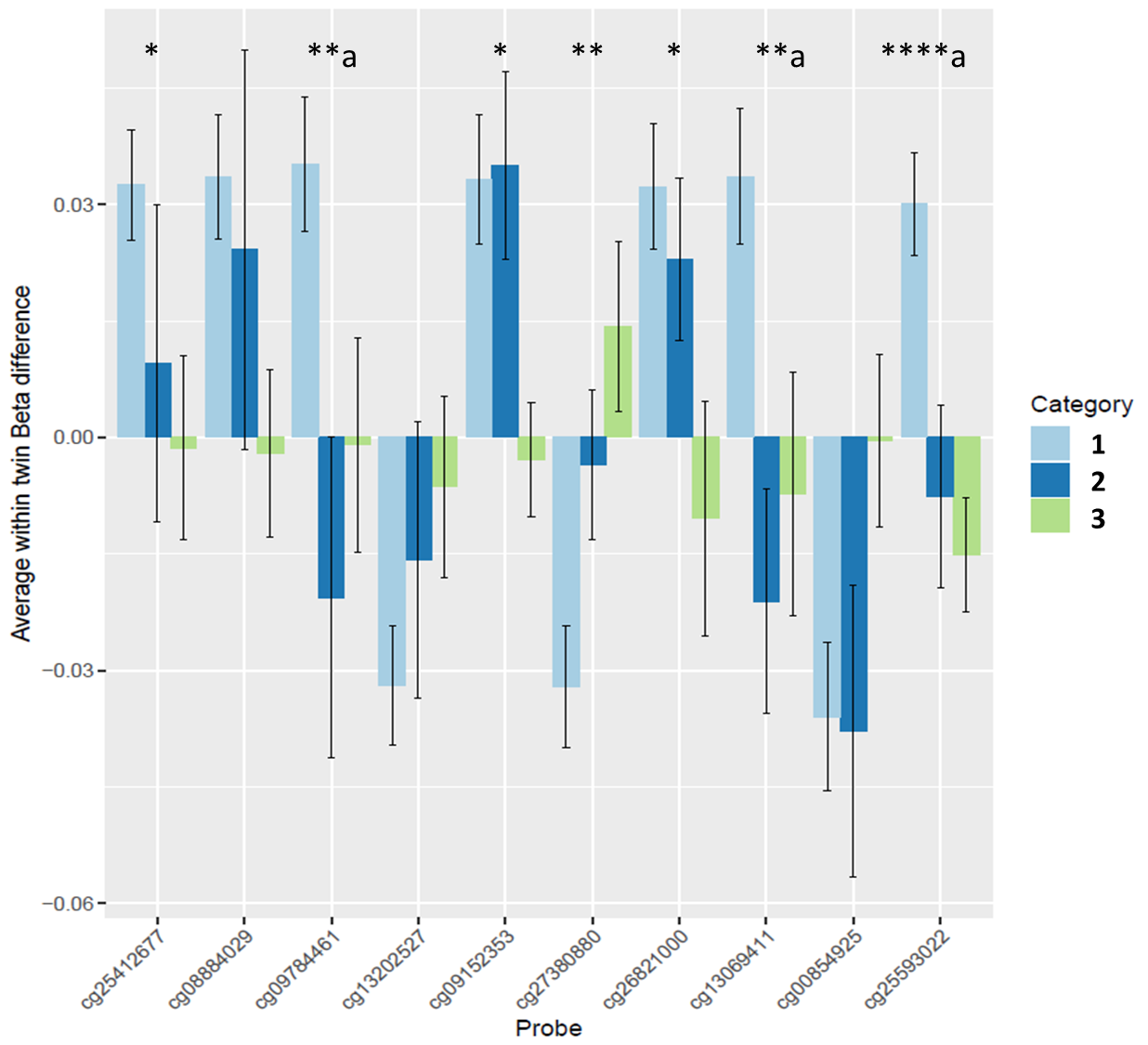
**

**Supplementary Figure 6b. Graph showing average within-twin β difference (∆β) in blood DNA at age 18 of adolescent victimization exposure-discordant monozygotic (MZ) twins (Group 1 = 41 pairs) and the age-matched concordant unexposed MZ twins (Group 2 = 12 pairs), and concordant age-matched exposed MZ twins (Group 3 = 22 pairs***). Note. Error bars represent +/- the standard error of the mean within-twin pair ∆β. ****P < 0.0001, ***P < 0.001, **P < 0.01, *P < 0.05; ^a^The average within-twin ∆β was greater in group 1 twins compared to both the group 2 and 3 twins.*

**
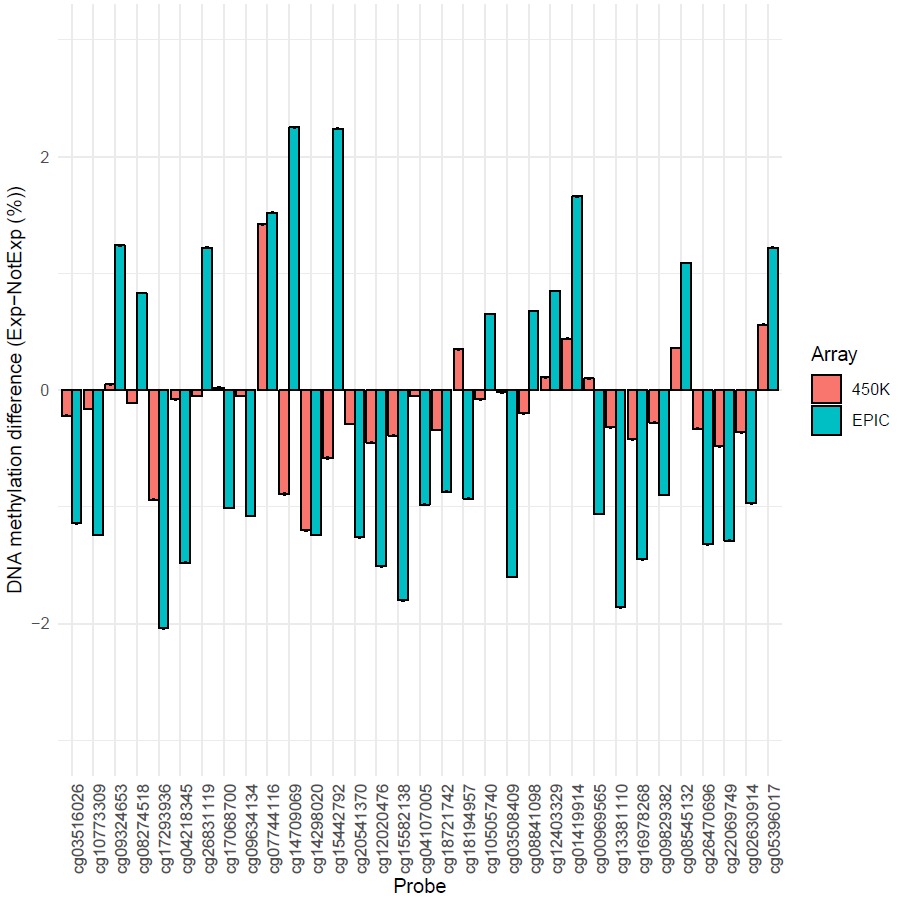
**

**Supplementary Figure 7. Comparison of effect sizes (y-axis) for the 33 severe adolescent victimization-associated overlapping probes in the age-18 blood epigenome-wide association study (x-axis) across the 450K and the EPIC array (N=233).**

**8a)**

**
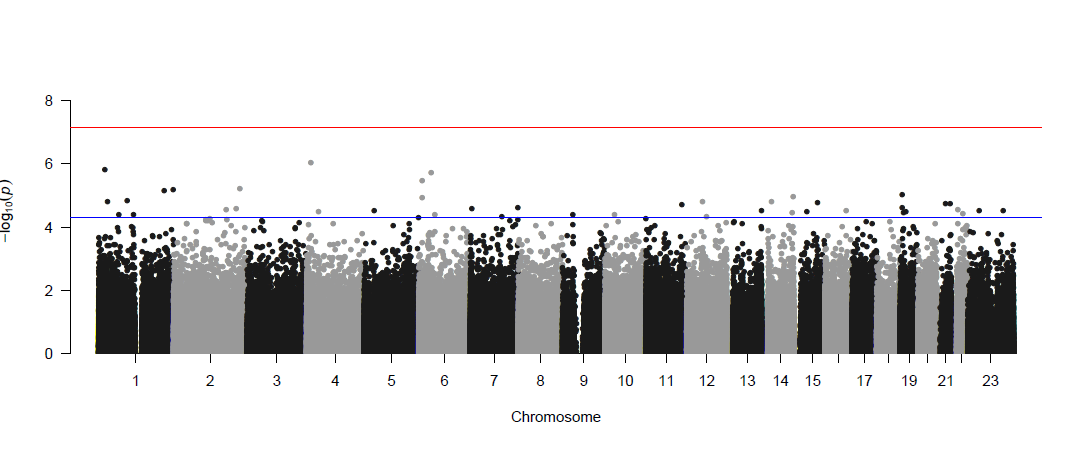
**

**8b)**

**
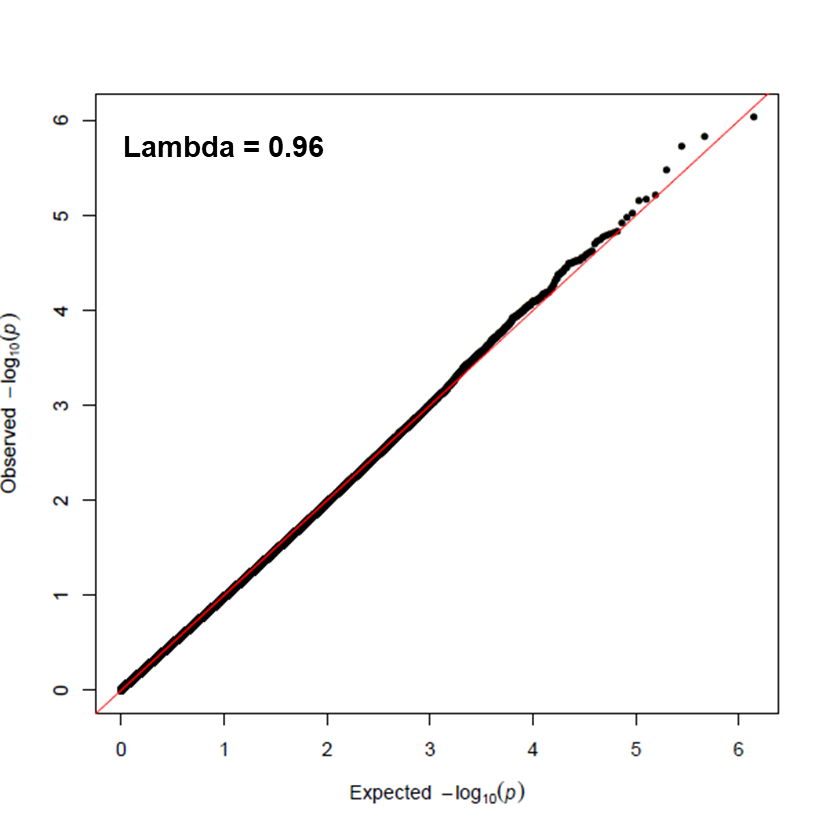
**

**Supplementary Figure 8. (a) Manhattan plot and (b) QQplot showing *P*-values from a linear regression with cluster-robust standard errors used to identify differential methylation associated with severe adolescent victimization exposure in the age-18 buccal epigenome-wide association study (red line – EPIC array *P*-value threshold of 7.2e-08 and blue line - Discovery *P*-value threshold of 5e-05).** The inflation factors λ was 0.96, indicating minimal evidence for genomic deflation/inflation. *Note.* *Covariates included gender, smoking pack years, and cell-type proportions***.**


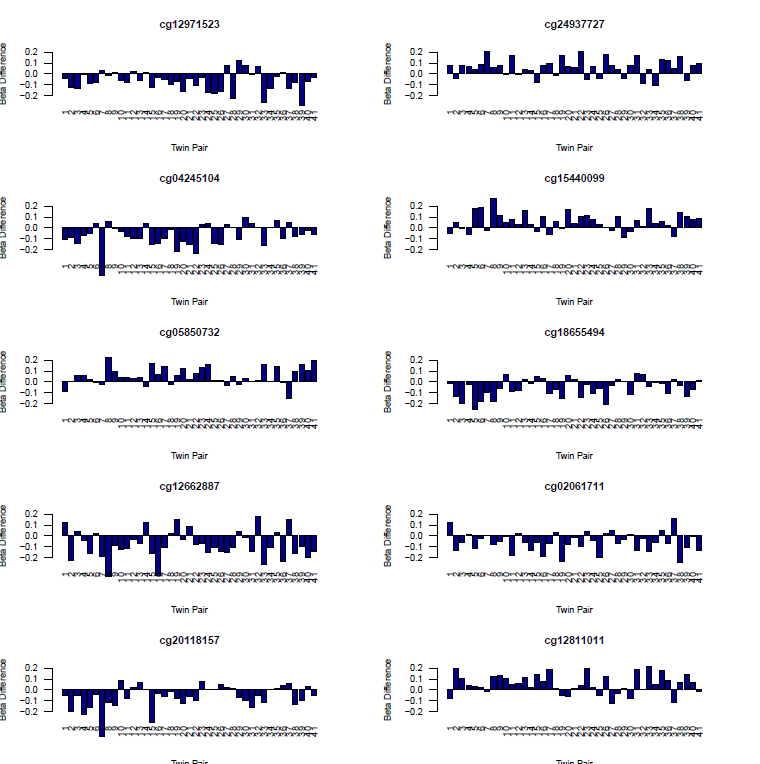


**Supplementary Figure 9a. Graphs showing the difference in DNA methylation (∆β) at age 18 between each pair of monozygotic (MZ) twins discordant for adolescent victimization at age 18 (exposed twin – unexposed co-twin) for each of the 10 top-ranked probes in buccal samples.** Consistent within-twin pair differences in DNA methylation at age 18 are observed across discordant MZ twin pairs (41 pairs) at the 10 top-ranked differentially methylated positions (DMPs).


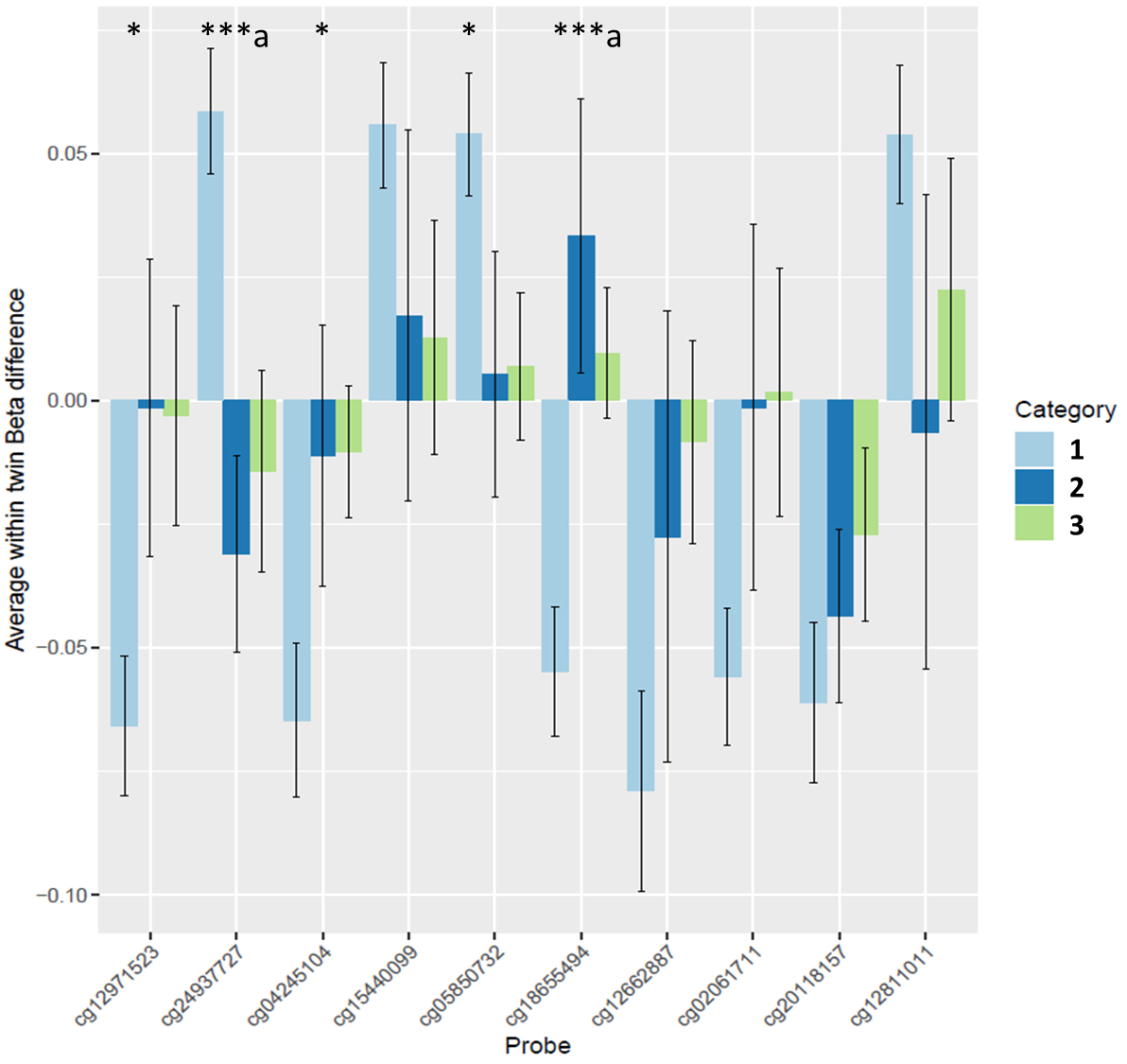


**Supplementary Figure 9b. Graph showing average within-twin β difference (∆β) in buccal DNA at age 18 of adolescent victimization exposure-discordant monozygotic (MZ) twins (Group 1 = 41 pairs) and the age-matched concordant unexposed MZ twins (Group 2 = 12 pairs), and concordant age-matched exposed MZ twins (Group 3 = 22 pairs).** *Note. Error bars represent +/- the standard error of the mean within-twin pair ∆β. ****P < 0.0001, ***P < 0.001, **P < 0.01, *P < 0.05; ^a^The average within-twin ∆β was greater in group 1 twins compared to both the group 2 and 3 twins.*
