# Supplementary material for: DNA methylation signatures of adolescent victimization: analysis of a longitudinal monozygotic twin sample
Source: Epigenetics. 2020 Dec 29;16(11):1169–86. doi: 10.1080/15592294.2020.1853317 (PMC8813077; doi:10.1080/15592294.2020.1853317)
Supplement: Supplemental Material [file KEPI_A_1853317_SM3941.zip › Supplementary Methods_revised_final.docx]

**Supplementary Methods**

***Study Cohort***

Participants were members of the Environmental Risk (E-Risk) Longitudinal Twin Study, which tracks the development of a nationally-representative birth cohort of 2232 British twin children. The sample was drawn from a larger cohort of twins born in England and Wales in 1994-1995 (1). Full details about the sample are reported elsewhere (2). Briefly, the E-Risk sample was constructed in 1999-2000, when 1116 families with same-sex 5-year-old twins (93% of those eligible) participated in home-visit assessments. Families were recruited to represent the UK population of families with newborns in the 1990s, based on residential location throughout England and Wales and mothers’ age. Teenaged mothers with twins were over-selected to replace high-risk families who were selectively lost to the register through non-response. Older mothers having twins via assisted reproduction were under-selected to avoid an excess of well-educated older mothers. E-Risk families are representative of UK households across the spectrum of neighborhood-level deprivation: 25.6% of E-Risk families live in “wealthy achiever” neighborhoods compared to 25.3% of households nation-wide; 5.3% vs 11.6% live in “urban prosperity” neighborhoods; 29.6% vs 26.9% live in “comfortably off” neighborhoods; 13.4% vs 13.9% live in “moderate means” neighborhoods; and 26.1% vs 20.7% live in “hard-pressed” neighborhoods (3, 4). E-Risk families under-represent “urban prosperity” neighborhoods because such households are likely to be childless. The sample comprised 56% monozygotic and 44% dizygotic twin pairs, and sex was evenly distributed within zygosity (49% male). All families were English speaking, and the majority (93.7%) were White.

Follow-up home visits were conducted when children were 7 years (98% of the 1116 E-Risk Study families participated), 10 years (96% participation), 12 years (96% participation) and 18 years (93% participation). Home visits at ages 5, 7, 10, and 12 years included assessments with participants as well as their mother (or primary caretaker); the home visit at age 18 included interviews only with the participants. Each twin participant was assessed by a different interviewer. The average age of the twins at the time of the age 18 assessment was 18.4 years (*SD*=0.36); all interviews were conducted after the 18^th^ birthday. There were no differences between those who did and did not take part at age 18 in terms of socioeconomic status (SES) assessed when the cohort was initially defined (χ^2^=0.86, *p*=0.65), age-5 IQ scores (*t*=0.98, *p*=0.33), or age-5 internalizing or externalizing behavior problems (*t*=0.40, *p*=0.69 and *t*=0.41, *p*=0.68, respectively). The Joint South London and Maudsley and the Institute of Psychiatry Research Ethics Committee approved each phase of the study. Parents gave informed consent, and participants gave assent at ages 5-12 and informed consent at age 18.

***Assessment of victimization in childhood***

Exposure to several types of victimization was assessed repeatedly when the children were 5, 7, 10, and 12 years of age (the age 5 assessment included exposure since birth) and dossiers have been compiled for each child with cumulative information about exposure to domestic violence between the mother and her partner; frequent bullying by peers; physical maltreatment by an adult; sexual abuse; emotional abuse; and neglect. The E-Risk team has previously reported evidence on the reliability and validity of the measures of domestic violence (5), bullying (6, 7), physical maltreatment and sexual abuse (8), emotional abuse (9), and physical neglect (10). All the component measures are outlined briefly below.

*Physical Domestic Violence.* Mothers reported about perpetration of and victimization by 12 forms of physical violence (e.g., slapping, hitting, kicking, strangling) from the Conflict Tactics Scale (11), on three assessment occasions during the child's first decade of life (when the children were 5, 7, and 10 years of age). Reports of either perpetration or victimization constituted evidence of physical domestic violence. Families in which no physical violence took place were coded as 0 (55.2%); families in which physical violence took place on one occasion were coded as 1 (28.0%); and families in which physical violence took place on multiple occasions were coded as 2 (16.8%).

*Bullying by Peers.* Experiences of victimization by bullies were assessed using both mothers’ and children’s reports. During the interview, the following standard definition of bullying was read out: *“Someone is being bullied when another child (a) says mean and hurtful things, makes fun, or calls a person mean and hurtful names; (b) completely ignores or excludes someone from their group of friends or leaves them out on purpose; (c) hits, kicks, or shoves a person, or locks them in a room; (d) tells lies or spreads rumours about them; and (e) other hurtful things like these. We call it bullying when these things happen often, and when it is difficult to make it stop. We do not call it bullying when it is done in a friendly or playful way.”* Mothers were interviewed when children were 7, 10, and 12 years old and asked whether either twin had been bullied by another child, responding never, yes, or frequently. We combined mothers’ reports when children were age 7 and 10 to derive a measure of victimization during primary school. Mothers’ reports when the children were 12 years old indexed victimization during secondary school. During private interviews with the children when they were 12 years old, the children indicated whether they had been bullied by another child during primary or secondary school. When a mother or a child reported victimization, the interviewer asked them to describe what happened. Notes taken by the interviewers were later checked by an independent rater to verify that the events reported could be classified as instances of bullying operationally defined as evidence of (a) repeated harmful actions, (b) between children, and (c) where there is a power differential between the bully and the victim (12). Although inter-rater reliability between mothers and children was only modest (*kappa* = 0.20-0.29), reports of victimization from both informants were similarly associated with children’s emotional and behavioural problems, suggesting that each informant provides a unique but meaningful perspective on bullying involvement (7). We thus combined mother and child reports of victimization to capture all instances of bullying victimization for primary and secondary school separately: reported as not victimized by both mother and child; reported by either mother or child as being occasionally victimized; and reported as being occasionally victimized by both informants or as frequently victimized by either mother or child or both (13). We then combined these primary and secondary school ratings to create a bullying victimization variable for the entire childhood period (5-12 years). Children who were never bullied in primary or secondary school or occasionally bullied during one of these time periods were coded as 0 (55.5%); children who were occasionally bullied during primary and secondary school, or frequently bullied during one of these time periods were coded as 1 (35.6%); and children who were frequently bullied at both primary and secondary school were coded as 2 (8.9%).

*Physical and sexual harm by an adult.* We assessed childhood physical and sexual harm in the E-Risk Study using an approach that resembles the process undertaken by child protection agencies. Essentially this is a two-stage process. In child protection, professionals such as teachers working with children typically raise concerns if they observe signs or symptoms or if they become aware of risk that children are victims of violence. When concerns are raised, child protection officers then review the concerns and evaluate them in the context of information previously gathered on that child or family in order to determine the likelihood that abuse has taken place. In the E-Risk Study, research workers visited the home in pairs, and were extensively trained to detect signs of abuse or neglect. Each time the two research workers visited a home, they interviewed the mother using a structured interview about child harm, tested the children, and observed the family environment using the Home Observation for Measurement of the Environment (HOME) (14). If either research worker had any concerns, they flagged up the case for review. Immediately after each home visit, a review was performed if a family was flagged. In addition, at each wave, any family who had been flagged on a prior wave of the study was automatically reviewed again. The reviews were performed independently by at least 2 clinical psychologists or psychiatrists, and were based on comprehensive dossiers compiled across multiple home visits for each study member during the course of the ongoing longitudinal study.

At age 5, assessments were based on the standardised clinical protocol from the MultiSite Child Development Project (12, 15). At ages 7, 10, and 12 this interview was modified to expand its coverage of contexts for child harm. Interviews were designed to enhance mothers’ comfort with reporting valid child maltreatment information, while also meeting researchers’ responsibilities for referral under the UK Children Act. Specifically, mothers were asked whether either of their twins had been intentionally harmed (physically or sexually) by an adult or had contact with welfare agencies. If caregivers endorsed a question, research workers made extensive notes on what had happened, and indicated whether physical and/or psychological harm had occurred. Under the U.K. Children Act, our responsibility was to secure intervention if maltreatment was current and ongoing. Such intervention on behalf of E-Risk families was carried out with parental cooperation in all but one case. No families left the study following intervention.

Over the years of data collection, the study developed a cumulative profile for each child, comprising the caregiver reports, recorded debriefings with research workers who had coded any indication of maltreatment at any of the successive home visits, recorded narratives of the successive caregiver interviews, and information from clinicians whenever the Study team made a child-protection referral. Each time we visited a home, the research workers flagged concerns, and if there was sufficient evidence to code definite harm then, we did so. If evidence only met the level of probable harm, we kept an “ongoing concern list” and if, at a later wave, there was continued evidence of probable harm, or new evidence, the code was upgraded to definite harm. The profiles were reviewed at the end of the age-12 phase by at least two clinical psychologists or psychiatrists. Initial inter-rater agreement between the coders was 90% in cases for whom maltreatment was identified (100% for cases of sexual abuse), and discrepantly coded cases were resolved by consensus review. These were coded as: 0 = no physical harm at any age; 1 = probable physical harm at any age; and 2 = definite physical harm at any age. There were 15.0% of children coded as probably being exposed to physical harm and 5.1% as definitely physically harmed by 12 years of age. There were 1.5% of the children coded as being exposed to sexual abuse.

*Emotional abuse and neglect.* These forms of maltreatment were coded from research workers’ narratives of the home visits at ages 5, 7, 10, and 12. We coded quite severe examples of parental behavior observed. For example, a mother who had schizophrenia screamed and swore at the children throughout the home visit. As another example, a father who was drunk during the home visit repeatedly spoke abusively to the children in front of the research workers. We found that coders could not empirically separate emotional abuse and emotional neglect in a reliable way and thus such experiences were coded together as emotional abuse/neglect. Inter-rater agreement between the coders exceeded 85% for cases with emotional abuse and neglect, and discrepant cases were resolved by consensus review. Children with no evidence of emotional abuse/neglect were coded as 0 (88.3%), those where there was some indication of emotionally inappropriate/potentially abusive or neglectful behavior were coded as 1 (8.7%), and where there was evidence of severe emotional abuse/neglect the children were coded as 2 (3.0%).

*Physical neglect.* The cumulative observations of the physical state of the home environment documented by the research workers during home visits to the twins at ages 5, 7, 10 and 12 were reviewed by two raters for evidence of physical neglect. This was defined as any sign that the caretaker was not providing a safe, sanitary, or healthy environment for the child. This included the child not having proper clothing or food, as well as grossly unsanitary home environments. (However, this did not include a family living in a crime-ridden neighborhood for economic reasons.) Inter-rater agreement between the coders exceeded 85%, and discrepantly coded cases were resolved by consensus review. Children with no evidence of physical neglect were coded as 0 (90.9%), those for whom there was an indication of minor physical neglect were coded as 1 (7.1%), and where there was evidence of severe physical neglect the children were coded as 2 (2.0%).

*Any severe victimization.* In this analysis we focused on whether children had one or more severe victimization experiences (i.e., those that received a code of ‘2’): 73.5% of children had zero victimization experiences; 26.5% had 1 or more severe victimization experiences by age 12.

***Assessment of victimization in adolescence***

We have previously reported evidence on the reliability and validity of our measurement of adolescent victimization (10). Here we summarize the method. Participants were interviewed about experiences between 12-18 years using the Juvenile Victimization Questionnaire (JVQ) (16, 17) adapted as a clinical interview. The JVQ has good psychometric properties (18) and was used in the U.K. National Society for the Prevention of Cruelty to Children national survey (19, 20), thereby providing benchmark values for comparisons with our cohort.

Within each pair of twins in our cohort, co-twins were interviewed separately by a different research worker and were assured of the confidentiality of their responses. The participants were advised that confidentiality would only be broken if they told the research worker that they were in immediate danger of being hurt, and in such situations the project leader would be informed and would contact the participant to discuss a plan for safety. We assessed 7 different forms of victimization: maltreatment, neglect, sexual victimization, family violence, peer/sibling victimization, cyber-victimization, and crime victimization. Each JVQ question was asked for the period ‘since you were 12’. Participants were given the option to say “yes” or “no” as to whether each type of victimization had occurred in the reporting period. Research workers could rate each item “maybe” if the participant seemed unsure or hesitant in their response or they were not convinced that the participant understood the question or was paying attention. Items rated as “maybe” were recoded as “no” or “yes” by the rating team based on the notes provided by the research workers. When insufficient notes were available, these responses were recoded conservatively as a “no”. Consistent with the JVQ manual, (16, 17) participants were coded as 1 if they reported any experience within each type of victimization category, or 0 if none of the experiences within the category were endorsed. If an experience was endorsed within a victimization category, follow-up questions were asked concerning how old the participant was when it (first) happened, whether the participant was physically injured in the event, whether the participant was upset or distressed by the event; and how long it went on for (by marking the number of years on a Life History Calendar (21). In addition, the interviewer wrote detailed notes based on the participant’s description of the worst event. If multiple experiences were endorsed within a victimization category, the participant was asked to identify and report about their worst experience.

All information from the JVQ interview was compiled into victimization dossiers. Using these dossiers, each of the seven victimization categories was rated by an expert in victimology and 3 other members of the E-Risk team who were trained on using the rating criteria. Ratings were made using a 6-point scale: 0 = not exposed, then 1-5 for increasing levels of severity. The anchor points for these ratings were adapted from the coding system used for the Childhood Experience of Care and Abuse interview (CECA) (22,23), which has good inter-rater reliability (22, 24). The CECA is a comprehensive semi-structured interview whose standardized coding system attempts to improve the objectivity of ratings by basing them on the coder’s perspective (rather than relying on the participant’s judgment) and focusing on concrete descriptions rather than perceptions or emotional responses to the questions, together with considering the context in which the adverse experience occurred.

In our adapted coding scheme, the anchor points of the scale differ for each victimization category, with some focused more on the severity of physical injury that is likely to have been incurred during victimization exposure (crime victimization, family violence, maltreatment), while others are more focused on the frequency of occurrence of victimization (peer/sibling victimization and cyber-victimization), the physical intrusiveness of the event (sexual victimization), or the pervasiveness of the effects of victimization (neglect). This reflects the different ways in which severity has previously been defined for different types of victimization (22, 25). (Given that our sample comprises twins, we also coded if any of the victimization events experienced by each twin had been perpetrated by their co-twin, as it is possible that growing up with a genetically related, same-age child could increase or decrease sibling victimization rates.) Each twin’s dossier was evaluated separately, and we did not use information provided in the co-twin’s dossier about their own or shared victimization experiences to rate direct or witnessed violence exposure for the target twin. High levels of inter-rater reliability were achieved for the severity ratings for all forms of victimization: crime victimization (intra-class correlation coefficient [ICC] = 0.89, p < 0.001), peer/sibling victimization (ICC = 0.91, p < 0.001), cyber-victimization (ICC = 0.90, p < 0.001), sexual victimization (ICC = 0.87, p < 0.001), family violence (ICC = 0.93, p < 0.001), maltreatment (ICC = 0.90, p < 0.001), and neglect (ICC = 0.74, p < 0.001).

The ratings for each type of victimization were then grouped into three classes: 0 – no exposure (score of 0), 1 – some exposure (score of 1, 2 or 3), and 2 – severe exposure (score of 4 or 5) due to small numbers for some of the rating points. Combining ratings of 4 and 5 is also consistent with previous studies using the CECA, which have collapsed comparable scale values to indicate presence of ‘severe’ abuse (22, 24, 26, 27). An adolescent was considered to have been exposed to severe victimization if any of the victimization experiences received a code of ‘4’ or ‘5’ (i.e., severe exposure): 64.6% of adolescents had zero severe victimization experiences; 35.4% had 1 or more severe victimization experiences.

***Quality control of DNA methylation data***

Signal intensities for all the samples (blood and buccal) were imported into the R programming environment (version 3.4.3) (28) using the methylumIDAT() function in the methylumi package (29) and the blood and buccal samples were processed together. A stringent quality control (QC) analysis was performed to check the quality of the data and remove poor quality samples from further analyses. The QC pipeline included the following steps:

1) excluding samples where either the median methylated or unmethylated intensity values were < 1500 (N=178);

2) using the control probes to ensure the sodium bisulphite conversion was successful, excluding any samples with a median score < 80 (N=10), as suggested by R wateRmelon (30);

3) using the clusterGender() function to perform Principal Component Analysis (PCA) on all probes and confirm sample sex match and exclude mismatched samples (N=6);

4) using the 59 SNP genotyping probes on the array with SNP array data to check DNA identity (N=14);

5) using the 59 SNP genotyping probes on the EPIC array to perform genetic correlations between the samples to confirm twin samples were from the same family and longitudinal DNA samples from the same individual (excluded N=0);

6) using the pfilter() function in R wateRmelon (30) to exclude a) samples with more than 5% of probes with a detection P value > 0.05, b) probes characterized by > 5% of samples with a detection P value > 0.05, and c) probes that have >5% of samples with a bead count <3 (excluded N = 0). Normalisation of the DNA methylation data was performed used the dasen() function in the wateRmelon package (30). Furthermore, blood cell counts were quantified for the age-18 blood samples using the estimateCellCounts() function from the minfi package.

7) excluding probes with common SNPs (>5% minor allele frequency) within 10bp of the single base extension (N=10,888), and probes with sequences previously identified as potentially hybridizing to multiple genomic loci were excluded (31) (N=44,210) and underperforming probes included in the Illumina Product Quality Notification PQN0223 dated 19 April 2017 (N=977).

8) removing non-variable probes showing minimal inter-individual variation (N=1,02,213) and retaining only probes where the range of DNA methylation values for the middle 80% of individuals was greater than 5% (32).

In total, 736/944 samples (80.0%) and 6,95,834 probes passed all QC steps and were included in subsequent analyses. Failed samples include one blood sample and 207 buccal samples.

***Comparison of the effect size of the severe adolescent victimization-associated probes quantified using 450K and EPIC array in overlapping E-Risk age-18 blood samples***

Raw intensity data (IDAT) files for both the 450K and EPIC data were loaded and combined into an array including only the overlapping probes (N= 453152) and subjects with age-18 blood DNA analysed on both the arrays (N=233). DNA methylation data was processed using wateRmelon package and the QC steps used in this study followed by normalization using *dasen()* function. Unreliable probes, SNP probes and cross-hybridising probes were removed, and remaining probes were used for further analysis (N=417658). Linear regression with clustered robust standard errors (33) was used to assess the association of the 33 (out of 72, *P*<5e-05) severe adolescent victimization-associated probes common across the age 18- blood EPIC and 450K arrays. The model was adjusted for gender, cell-type composition and smoking pack years similar to the current study. A correlation analysis was performed using cor.test () function in R to assess the correlation between the effect sizes for these 33 (out of 72) severe adolescent victimization-associated overlapping probes across the EPIC and 450K arrays. An exact binomial test (binom.test in R) was used to test whether the directional consistency between the two arrays was observed more often than expected by chance. For assessing the reproducibility between the two arrays, we also calculated correlation coefficients as well as variance of β-methylation per probe, i.e., for individual probes across all subjects. The correlation coefficients and probe variance were generated for the EPIC and 450K datasets normalized together as well as based on normalization carried out in the respective studies to assess the effect of normalization technique on the methylation values.

**REFERENCES**

1. Trouton A, Spinath, F.M. and Plomin, R. (2002) Twins early development study (TEDS): a multivariate, longitudinal genetic investigation of language, cognition and behavior problems in childhood. *Twin Res* **5**, 444-448.

2. Moffitt, T.E., E-Risk Study Team. (2002) Teen-aged mothers in contemporary Britain. *J Child Psychol Psychiatry* **43**, 727-742.

3. CACI. (2006) ACORN user guide. London: CACI Information Services.

4. Caspi, A., Taylor, A., Moffitt, T.E. and Plomin, R. (2000) Neighborhood deprivation affects children's mental health: environmental risks identified in a genetic design. *Psychol Sci* **11**, 338-342.

5. Moffitt, T.E., Caspi, A., Krueger, R.F., Magdol, L., Margolin, G., Silva, P.A. and Sydney, R. (1997) Do partners agree about abuse in their relationship?: A psychometric evaluation of interpartner agreement. *Psychol Assess*, **9**, 47-56.

6 Arseneault, L., Walsh, E., Trzesniewski, K., Newcombe, R., Caspi, A. and Moffitt, T.E. (2006) Bullying victimization uniquely contributes to adjustment problems in young children: a nationally representative cohort study. *Pediatrics*, **118**, 130-138.

7 Shakoor, S., Jaffee, S.R., Andreou, P., Bowes, L., Ambler, A.P., Caspi, A., Moffitt, T.E. and Arseneault, L. (2011) Mothers and children as informants of bullying victimization: results from an epidemiological cohort of children. *J Abnorm Child Psychol*, **39**, 379-387.

8 Jaffee, S.R., Caspi, A., Moffitt, T.E. and Taylor, A. (2004) Physical maltreatment victim to antisocial child: evidence of an environmentally mediated process. *J Abnorm Psychol*, **113**, 44-55.

9 Danese, A., Moffitt, T.E., Arseneault, L., Bleiberg, B.A., Dinardo, P.B., Gandelman, S.B., Houts, R., Ambler, A., Fisher, H.L., Poulton, R. *et al.* (2017) The origins of cognitive deficits in victimized children: implications for neuroscientists and clinicians. *Am J Psychiatry*, **174**, 349-361.

10 Fisher, H.L., Caspi, A., Moffitt, T.E., Wertz, J., Gray, R., Newbury, J., Ambler, A., Zavos, H., Danese, A., Mill, J. *et al.* (2015) Measuring adolescents' exposure to victimization: The Environmental Risk (E-Risk) Longitudinal Twin Study. *Dev Psychopathol*, **27**, 1399-1416.

11 Straus, M.A. (1990) Straus, M.A. and Gelles, R.G. (eds.), In *Physical violence in American families: Risk factors and adaptations to violence in 8,145 families*. Transaction Press, New Brunswick, NJ, pp. 403-424.

12 Lansford, J.E., Dodge, K.A., Pettit, G.S., Bates, J.E., Crozier, J. and Kaplow, J. (2002) A 12-year prospective study of the long-term effects of early child physical maltreatment on psychological, behavioral, and academic problems in adolescence. *Arch Pediatr Adolesc Med*, **156**, 824-830.

13 Bowes, L., Maughan, B., Ball, H., Shakoor, S., Ouellet-Morin, I., Caspi, A., Moffitt, T.E. and Arseneault, L. (2013) Chronic bullying victimization across school transitions: the role of genetic and environmental influences. *Dev Psychopathol*, **25**, 333-346.

14 Bradley, R.H. and Caldwell, B.M. (1977) Home observation for measurement of the environment: a validation study of screening efficiency. *Am J Ment Defic*, **81**, 417-420.

15 Dodge, K.A., Bates, J.E. and Pettit, G.S. (1990) Mechanisms in the cycle of violence. *Science*, **250**, 1678-1683.

16 Finkelhor, D., Hamby, S.L., Turner, H. and Ormrod, R. (2011) *The Juvenile Victimization Questionnaire: 2nd Revision (JVQ-R2)*. Crimes Against Children Research Center, Durham, NH.

17 Hamby, S.L., Finkelhor, D., Ormrod, R. and Turner, H. (2004) *The Juvenile Victimization Questionnaire (JVQ): Administration and Scoring Manual*. Crimes Against Children Research Centre, Durham, NH.

18 Finkelhor, D., Hamby, S.L., Ormrod, R. and Turner, H. (2005) The Juvenile Victimization Questionnaire: reliability, validity, and national norms. *Child Abuse Negl*, **29**, 383-412.

19 Radford, L., Corral, S., Bradley, C., Bassett, C., Howat, N. and Collishaw, S. (2011). *Child abuse and neglect in the UK today*. National Society for the Prevention of Cruelty to Children, London: U.K.

20 Radford, L., Corral, S., Bradley, C. and Fisher, H.L. (2013) The prevalence and impact of child maltreatment and other types of victimization in the UK: findings from a population survey of caregivers, children and young people and young adults. *Child Abuse Negl*, **37**, 801-813.

21 Caspi, A., Moffitt, T.E., Thornton, A. and Freedman, D. (1996) The life history calendar: A research and clinical assessment method for collecting retrospective event-history data. *Int J Methods in Psychiatr Res*, **6**, 101-114.

22 Bifulco, A., Brown, G.W. and Harris, T.O. (1994) Childhood Experience of Care and Abuse (CECA): a retrospective interview measure. *J Child Psychol Psychiatry*, **35**, 1419-1435.

23 Bifulco, A., Brown, G.W., Neubauer, A., Moran, P.M. and Harris, T.O. (1994) *Childhood Experience of Care and Abuse (CECA) training manual*. Royal Holloway College, University of London, London.

24 Bifulco, A., Brown, G.W., Lillie, A. and Jarvis, J. (1997) Memories of childhood neglect and abuse: corroboration in a series of sisters. *J Child Psychol Psychiatry*, **38**, 365-374.

25 Barnett, D., Manly, J.T. and Cicchetti, D. (1993) Cicchetti, D. and Toth, S.L. (eds.), In *Child abuse, child develpment, and social policy*. Ablex, Norwood, NJ, pp. 7-74.

26 Bifulco, A., Brown, G.W., Moran, P., Ball, C. and Campbell, C. (1998) Predicting depression in women: the role of past and present vulnerability. *Psychol Med*, **28**, 39-50.

27 Fisher, H.L., Bunn, A., Jacobs, C., Moran, P. and Bifulco, A. (2011) Concordance between mother and offspring retrospective reports of childhood adversity. *Child Abuse Negl*, **35**, 117-122.

28 R Core Team (2017) R: A Language and Environment for Statistical Computing.

29 Davis, S., Du, P., Bilke, S., Triche, T.J. and Bootwalla, M. (2015) *methylumi: Handle Illumina methylation data. R package version 2.14.0.*

30 Pidsley, R., Y., W.C.C., Volta, M., Lunnon, K., Mill, J. and Schalkwyk, L.C. (2013) A data-driven approach to preprocessing Illumina 450K methylation array data. *BMC Genomics*, **14**, 293.

31 McCartney, D.L., Walker, R.M., Morris, S.W., McIntosh, A.M., Porteous, D.J. and Evans, K.L. (2016) Identification of polymorphic and off-target probe binding sites on the Illumina Infinium MethylationEPIC BeadChip. *Genom Data*, **9**, 22-24.

32 Hannon, E., Knox, O., Sugden, K., Burrage, J., Wong, C.C.Y., Belsky, D.W., Corcoran, D.L., Arseneault, L., Moffitt, T.E., Caspi, A. *et al.* (2018) Characterizing genetic and environmental influences on variable DNA methylation using monozygotic and dizygotic twins. *PLoS Genet*, **14**, e1007544.

33 Staley, J.R., Suderman, M., Simpkin, A.J., Gaunt, T.R., Heron, J., Relton, C.L. and Tilling, K. (2018) Longitudinal analysis strategies for modelling epigenetic trajectories. *Int J Epidemiol*, **47**, 516-525.
